# Polar Desolvation and Position 226 of Pancreatic and Neutrophil Elastases Are Crucial to their Affinity for the Kunitz-Type Inhibitors ShPI-1 and ShPI-1/K13L

**DOI:** 10.1371/journal.pone.0137787

**Published:** 2015-09-15

**Authors:** Jorge Enrique Hernández González, Rossana García-Fernández, Pedro Alberto Valiente

**Affiliations:** Centro de Estudios de Proteínas, Facultad de Biología, Universidad de La Habana, La Habana, Cuba; University of Copenhagen, DENMARK

## Abstract

The Kunitz-type protease inhibitor ShPI-1 inhibits human neutrophil elastase (HNE, *K*_*i*_ = 2.35·10^−8^ M) but does not interact with the porcine pancreatic elastase (PPE); whereas its P1 site variant, ShPI-1/K13L, inhibits both HNE and PPE (*K*_*i*_ = 1.3·10^−9^ M, and *K*_*i*_ = 1.2·10^−8^ M, respectively). By employing a combination of molecular modeling tools, e.g., structural alignment, molecular dynamics simulations and Molecular Mechanics Generalized-Born/Poisson-Boltzmann Surface Area free energy calculations, we showed that D226 of HNE plays a critical role in the interaction of this enzyme with ShPI-1 through the formation of a strong salt bridge and hydrogen bonds with K13 at the inhibitor’s P1 site, which compensate the unfavorable polar-desolvation penalty of the latter residue. Conversely, T226 of PPE is unable to establish strong interactions with K13, thereby precluding the insertion of K13 side-chain into the S1 subsite of this enzyme. An alternative conformation of K13 site-chain placed at the entrance of the S1 subsite of PPE, similar to that observed in the crystal structure of ShPI-1 in complex with chymotrypsin (PDB: 3T62), is also unfavorable due to the lack of stabilizing pair-wise interactions. In addition, our results suggest that the higher affinity of ShPI-1/K13L for both elastases mainly arises from the lower polar-desolvation penalty of L13 compared to that of K13, and not from stronger pair-wise interactions of the former residue with those of each enzyme. These results provide insights into the PPE and HNE inhibition and may contribute to the design of more potent and/or specific inhibitors toward one of these proteases.

## Introduction

Elastases constitute a group of serine proteases (SPs) considered as attractive therapeutic targets due to their involvement in different pathologic processes. For example, pancreatic elastase is associated with pancreatitis, whereas proteinase 3 and HNE (UNIPROTKB: P08246) are involved in rheumatoid arthritis as well as in respiratory and inflammatory diseases [1–6]. These findings have encouraged the search for endogenous inhibitors and the modification of protease inhibitors (PIs) to increase their activity against target enzymes or to study the protease-inhibitor interactions involved in complex formation [5, 7, 8].

PIs are widespread naturally-occurring molecules that regulate the enzymatic activity of proteases, thereby avoiding the unwanted proteolysis and guaranteeing the partial proteolysis as a physiological event [9, 10]. These molecules have been used as tools for structure-function studies with their target proteases, as well as in biotechnology and biomedicine [11]. The peptidic inhibitors belonging to the BPTI-Kunitz family are among the best characterized and largest group of PIs [12]. They mainly inhibit SPs and are classified as canonical inhibitors according to their interaction mechanism [10, 13, 14]. The bovine pancreatic trypsin inhibitor (BPTI, UNIPROTKB: P00974) is regarded as the prototypical molecule of the BPTI-Kunitz family and has been widely used as a model for protease-inhibitor interaction studies [15, 16].

**Fig 1:**
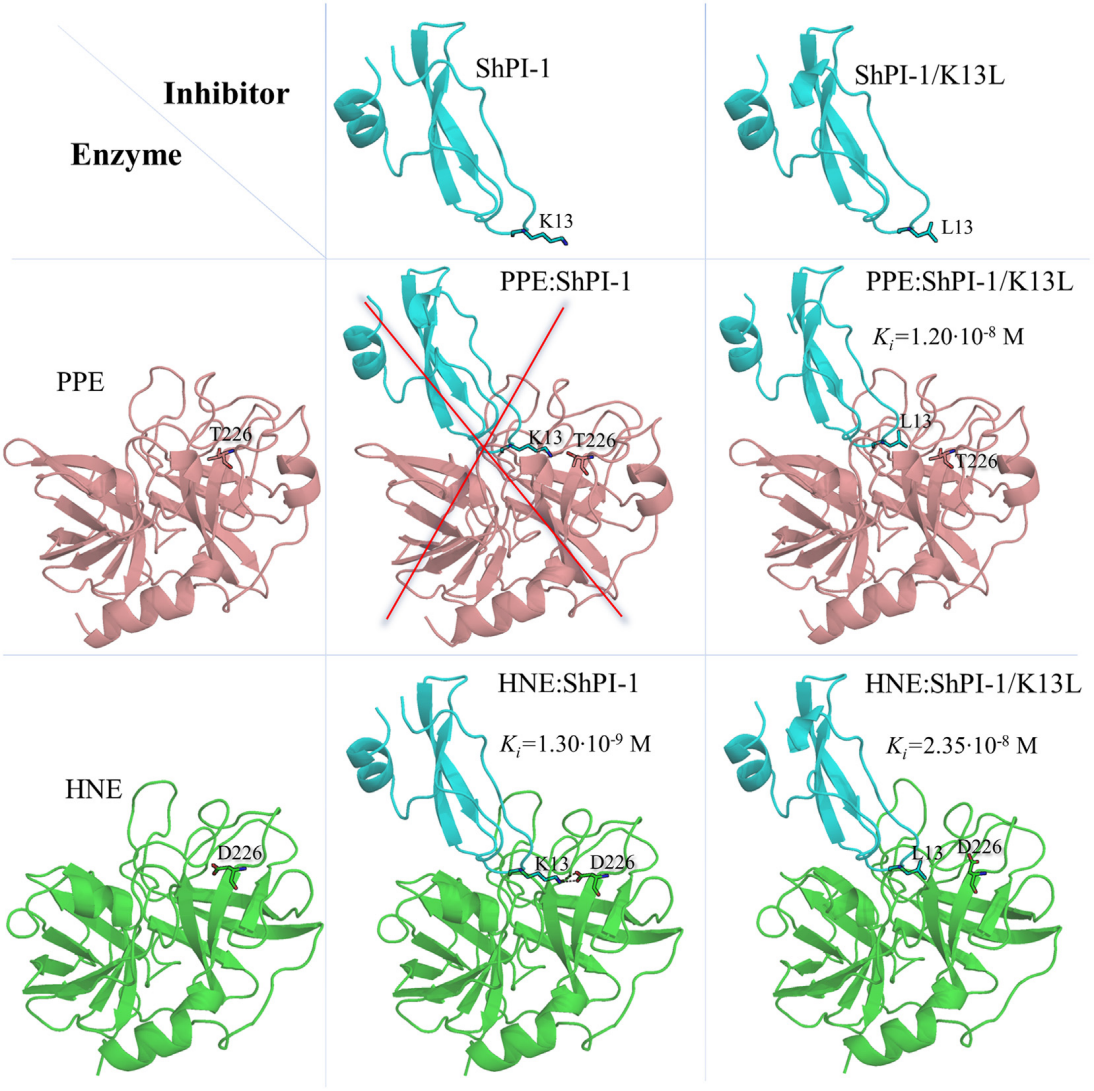
Overview of the Experimental Affinities and Main Structural Features of the Studied Complexes. The interaction of both elastases with ShPI-1 and ShPI-1/K13L is represented as a matrix-like scheme. The inability of ShPI-1 to interact with PPE is represented by a red cross on a hypothetical complex structure. Experimentally-determined *K*_*i*_ values for the other complexes are shown together with their respective 3D structures in cartoon representation. The crystal structure of PPE in complex with ShPI-1/K13L complex (PDB: 3UOU) was used as a template to model the structures of the remaining complexes (see Materials and Methods below). The HNE structure was extracted from the PDB 2Z7F. It is noteworthy that a hypothetical structure of the non-existing PPE:ShPI-1 complex was also generated to predict the underlying structural and energetic factors preventing its formation in solution. The P1 site residues (K13 and L13) and the residues at position 226 of both elastases (T226 and D226) are shown in stick representation.

The homologue inhibitor ShPI-1 (UNIPROTKB: P31713) is a 55 amino acid polypeptide (6110.6 Da) isolated from the sea anemone *Stichodactyla helianthus*. Its sequence identity with BPTI is ~34%, both of them bearing a basic Lys residue at the P1 site [17, 18]. The three-dimensional (3D) structure of ShPI-1 (PDB: 1SHP) shows the main structural features of BPTI-Kunitz domains [19]. ShPI-1 is a tight binding inhibitor of various SPs of the S1 family and is also active against proteases belonging to other mechanistic families, such as cysteine and aspartic proteases, an unusual behavior for most BPTI-Kunitz inhibitors [17, 18]. Recently, the 3D structure of the P1 site variant ShPI-1/K13L in complex with PPE (UNIPROTKB: P00772) (PDB: 3UOU) was determined by X-ray diffraction at a resolution of 2.00 Å (Fig 1) [7]. Remarkably, this is the only experimentally-determined 3D structure of an elastase-like SP in complex with a BPTI-Kunitz domain reported so far. According to the structure, ShPI-1/K13L binds PPE through the expected canonical binding mode, comprising residues from P6 to P5’ site of the inhibitor’s primary binding loop, as well as from P19’ to P24’ site of the secondary binding loop [7]. In addition, more than 30% of all contacts at the complex interface involve the P1 site residue (L13) and the enzyme residues within the complementary S1 subsite [7].

The functional characterization of a recombinant variant of wild-type ShPI-1 showed that this inhibitor is active against HNE (*K*_*i*_ = 2.35·10^−8^ M), but no inhibitory activity against PPE has been measured regardless the increase of the inhibitor concentration in the enzymatic assays (Fig 1) [7]. This behavior is qualitatively similar to that of BPTI, but certainly more pronounced, since the latter displays detectable ˗although low˗ inhibitory activities against both PPE (*K*_*i*_ = 1.0·10^−3^ M) and HNE (*K*_*i*_ = 3.5·10^−6^ M) [20]. Previous studies have shown that the S1 subsite of HNE is more flexible than that of PPE, which, in turn, favors its interaction with different residues at the P1 site [21, 22]. Moreover, it has been suggested that D226 at the S1 subsite of HNE might be involved in the stabilization of basic residues at the P1 site [7, 22], which would explain the higher specificity of ShPI-1 and other BPTI-Kunitz inhibitors toward HNE [7]. However, the energetic contribution of the interaction between D226 of HNE and a basic residue at the inhibitor’s P1 site has never been assessed before. On the other hand, the K13L amino acid substitution at the P1 site improved the inhibition of HNE (*K*_*i*_ = 1.3·10^−9^ M) and transformed ShPI-1 into a tight-binding inhibitor of PPE (*K*_*i*_ = 1.2·10^−8^ M) (Fig 1) [7]. Although previous works have demonstrated the preference of elastases for aliphatic residues at the inhibitor’s P1 site (see S1 Fig to check the overall hydrophobic nature of the S1 residues of HNE and PPE) [7, 23], the energetic basis of the improved inhibitory activity has not been elucidated.

In this manuscript, a detailed structural and energetic analysis of the binding of ShPI-1 and ShPI-1/K13L to PPE and HNE is presented by combining structural alignment of both elastases, molecular dynamics (MD) simulations and Molecular Mechanics Generalized-Born (Poisson-Boltzmann) Surface Area (MM-GB(PB)SA) free energy calculations [24–30]. These predictions showed that the interaction, i.e, salt bridge and hydrogen bonds, between K13 of ShPI-1 and D226 of HNE (Fig 1), is crucial to the complex formation. Accordingly, its abrogation through the *in silico* mutation D226A precludes the binding of ShPI-1 to the mutated enzyme. We also proposed that the presence of a Thr residue at the equivalent position of PPE (T226), which does not establish strong interactions with K13, greatly disfavors the binding of the wild-type inhibitor to this enzyme (Fig 1). Furthermore, it was predicted that the stronger interaction of both elastases with ShPI-1/K13L is largely caused by the lower polar-desolvation penalty of the L13 compared to that of K13, and not by stronger pair-wise interactions of the former with the residues at the S1 subsite of each enzyme. Overall, these results point out the importance of considering both pair-wise interactions and desolvation effects when analyzing the relative affinities of protein-protein complexes.

## Materials and Methods

### Prediction of protease-inhibitor 3D structures through structural alignment and *in silico* mutations

**Fig 2:**
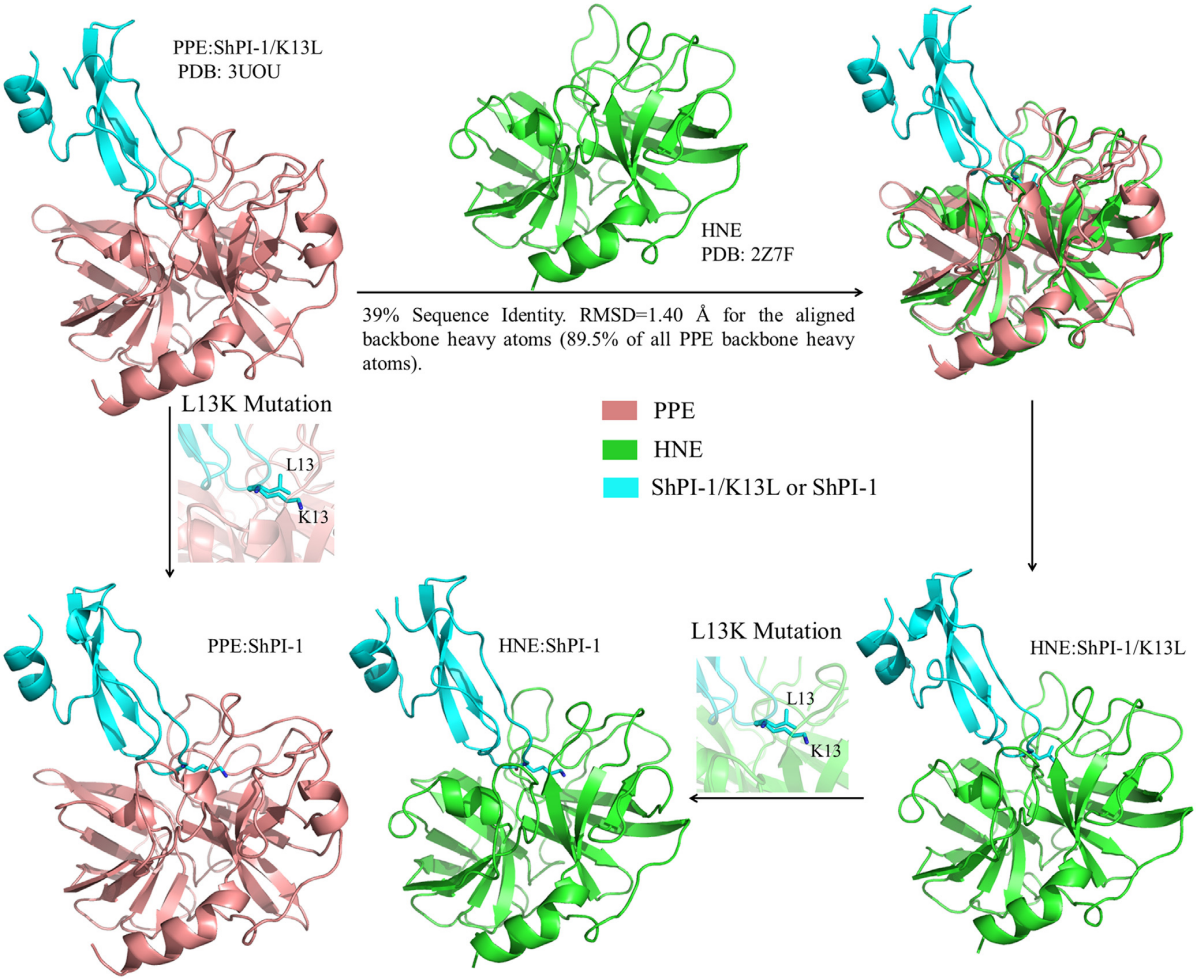
Workflow for the Generation of the 3D Models of HNE:ShPI-1/K13L, HNE:ShPI-1 and PPE:ShPI-1 Complexes. The structural alignment of PPE and HNE was performed with Modeller v9.5 [31, 32]. The *in silico* L13K point mutation on the inhibitor was carried out with Pymol v.1.7.0.0 [33].

Previous lines of evidence have demonstrated that the same binding mode is preserved in protein-protein complexes formed by close homologues with 30–40% sequence identity [25]. Therefore, the 3D structure of the PPE:ShPI-1/K13L complex (PDB:3UOU) was used here as a template to generate 3D models of HNE (~39% sequence identity with PPE, S1 Fig) in complex with both inhibitor variants, i.e., HNE:ShPI-1 and HNE:ShPI-1/K13L. Likewise, the 3D model of the complex between the wild-type inhibitor ShPI-1 and PPE was calculated in order to perform structural and energetic analyses, although previous functional studies have not detected the formation of this complex [7]. First, the 3D model of the HNE:ShPI-1/K13L complex was obtained by superimposing the HNE 3D structure extracted from the crystal structure of this protease in complex with the C-terminal domain of the secretory leukocyte protease inhibitor (PDB:2Z7F) onto the PPE chain of the template (structural alignment) using the program Modeller v9.5 (Fig 2) [31, 32]. Then, the 3D models of the PPE:ShPI-1 and HNE:ShPI-1 complexes were generated by performing the *in silico* K13L mutation with the mutagenesis tool of Pymol v1.7.0.0 (Fig 2) [33]. K13 rotamers were selected by visual inspection. This procedure was also employed for generating various alanine point mutations at the complex interfaces, which are required for computational alanine scanning (CAS) [28].

### Preparation of starting structures for energy minimization and molecular dynamics simulations

The protonation states of ionizable residues and His tautomers in the protease-inhibitor complexes were determined at pH = 7.4 with the program PDB2PQR (http://nbcr-222.ucsd.edu/pdb2pqr_1.8/), which uses PROPKA for the prediction of p*K*_*a*_ values [34]. All acid (Glu and Asp) and basic (Lys and Arg) residues were predicted in their respective ionic forms. His residues were always neuter and appeared either in the HID or in the HIE tautomeric form. HID tautomers were specifically predicted at positions 57, 200 and 210 of PPE, and 57 and 210 of HNE in all complexes; the remaining His residues of both enzymes being predicted as HIE tautomers. The tautomeric form of H57, a residue of the catalytic triad of SPs, was particularly checked, since the information for its protonation state is available [35]. The unique His residue of ShPI-1 and ShPI-1/K13L, i.e., H47, was predicted as HID tautomer, in agreement with the structure of ShPI-1 (PDB:1SHP) determined by Nuclear Magnetic Resonance [19]. The remaining steps necessary for energy minimization (EM) were performed with GROMACS v4.5.5 package [36]. Briefly, hydrogen atoms were added to the starting structures using the protonation states of ionizable residues predicted before. Disulfide (S-S) bonds between all Cys residue pairs were built in the correct topology. Then, a dodecahedral solvation box with edges spanning at least 1 nm from the solute surface was created around each complex. TIP3P water molecules were subsequently added and periodic boundary conditions (PBC) were settled in the limits of the solvation box. Electroneutrality was guaranteed by adding Na^+^ and Cl^−^ ions into the unit cells at the appropriate ratio to reach final NaCl concentrations of 0.50 mol/L and 0.05 mol/L, which are similar to those used during the experimental determination of *K*_*i*_ values against HNE and PPE, respectively [7].

### Energy minimization and molecular dynamics simulations

The protocol employed here to perform MD simulations involves prior EM and position-restrained equilibration, as outlined by Lindahl for lysozyme in water [37]. AMBER99SB force-field [38] was used for the calculation of forces during both EM and MD simulations, which were carried out with the mdrun program of GROMACS v4.5.5 [36]. All systems were subjected to 15000 steps of steepest descents minimization with an integration step of 0.1 nm. The maximum tolerance was set to 1000 kJ mol^-1^ nm^-1^. Cutoff radii of 1.4 nm and 1.0 nm were established for the calculation of van der Waals and short-range electrostatic interactions, respectively. The Particle Mesh Ewald algorithm was used to handle long-range electrostatic interactions [39]. Interatomic distances were left unconstrained in all systems during EM.

Subsequently, water molecules were relaxed around the complexes by 200 ps of position-restrained equilibration. Harmonic restraints with force constants of 1000 kJ mol^-1^ nm^-2^ were applied to all heavy atoms of the proteins. The treatment of van der Waals and electrostatic interactions was identical to that of EM. The Newton’s equation of motion was solved using the leap-frog algorithm, with and integration step of 2 fs [40]. Velocity rescaling [41] and Berendsen weak coupling [42] algorithms were used to keep temperature (*T*) and pressure (*p*) constant at 298 K and 1 atm, respectively. Interatomic distances were constrained by the Linear Constraints Solver algorithm [43] and random initial velocities obeying the Maxwell-Boltzmann distribution at 298 K were assigned to each atom prior to the MD simulations.

Finally, Langevin dynamics simulations [44] were carried out for each system during 25 ns at *T* = 298 K and *p* = 1 atm. The simulation time of a hypothetical non-existing PPE:ShPI-1 complex was particularly extended up to 125 ns to increase the probability of sampling the eventual disruption of the complex interface. The Parrinello-Rahman coupling algorithm [45, 46] was used to keep pressure constant and the friction coefficient (ξ) was set to 0.5 ps^-1^ in all systems, as recommended elsewhere [36]. The treatment of non-bonded interactions and constraints, as well as the integration step were identical to those used during the position-restrained MD simulations. Snapshots were saved at 10 ps intervals.

### Contact analysis at the complex interfaces

To obtain the interatomic contacts at the interfaces of the four complexes, their representative structures were calculated from the productive (frames collected after the equilibration time (*t*_*eq*_)) MD simulations by using clustering analysis with g_cluster (GROMACS v4.5.5). Van der Waals contacts between residues belonging to different protein chains were then defined using a cutoff distance of 4 Å. Additionally, hydrogen bonds were calculated using the g_hbond program (GROMACS v4.5.5), based on the following geometrical criteria: *i*) a distance ≤3.5 Å between the donor and the acceptor and *ii*) an acceptor-donor-hydrogen angle ≤30^○^. The time stability of hydrogen bonds was also assessed during MD simulations. Finally the formation of salt bridges between oppositely-charged residue pairs interacting within a distance of 4 Å at least in one snapshot was assessed with the salt bridge extension of Visual Molecular Dynamics v1.9.1 (VMD) [47]. The average distance between the oppositely-charged groups of the interacting residues was determined from the productive MD simulations and was used as a measure of salt bridge strength.

### Assessing the stability of the PPE:ShPI-1 and HNE:ShPI-1 complexes to a small interface disruption

As an alternative approach to study the differential interaction of ShPI-1 with both elastases, we assessed the stability of the starting 3D models of the PPE:ShPI-1 and HNE:ShPI-1 complexes to small disruptions of their respective interfaces. This task was accomplished by first moving the ShPI-1 molecule 3.2 Å away from the PPE molecule using VMD v1.9.1. After the perturbation, the amine group of K13 (P1) of ShPI-1 was placed at the entrance of the S1 subsite of PPE. Subsequently, the disrupted HNE:ShPI-1 complex was generated by superimposing the HNE structure onto that of PPE; thus, the same perturbed coordinates of ShPI-1 were used in both complexes. The time evolution of the disrupted systems was assessed by performing 60 ns MD simulations, following the previously-described steps and conditions. The structural changes during the MD simulations were determined by performing RMSD analysis and distance time profiles.

### MM-GB(PB)SA free energy calculations

The MM-GB(PB)SA method calculates the binding free energy (Δ*G*_*bind*_) as the sum of three components, i.e., the molecular energy in gas phase (Δ*E*_*gas*_), the solvation free energy (Δ*G*_*solv*_) and the entropy contribution (-*T∙*Δ*S*). When the third term is neglected, the computed value is that of the effective free energy (Δ*G*_*eff*_), which usually suffices for comparing the relative affinities of a series of similar ligands for a given receptor [30, 48]. The term Δ*E*_*gas*_, which includes the internal (Δ*E*_*int*_), the van der Waals (Δ*E*_*vw*_) and the electrostatic (Δ*E*_*el*_) energies, is derived from the force-field equations. The term Δ*G*_*solv*_ is further decomposed into two components, i.e., the polar-solvation free energy (Δ*G*_*GB/PB*_) and the non-polar solvation free energy (Δ*G*_*SA*_) [30, 48]. The former is calculated through different Generalized-Born (GB) or Poisson-Boltzmann (PB) implicit-solvation models, whereas the latter is obtained by the equation:

$$\Delta G_{SA}\left(X\right)=\gamma \Delta SA\left(X\right)+\beta$$ (1)

in which Δ*SA*(*X*) represents the solvent-accessible surface area variation of the solute molecule *X* upon complex formation, while *γ* and *β* are empiric constants whose values for GB models are almost always 0.0072 kcal·Å^-2^·mol^-1^ and 0, respectively [48, 49]. Finally, *T*·Δ*S* is frequently computed by normal-mode analysis and is, therefore, the most computationally-demanding step of the MM-GB(PB)SA method [48, 50].

The MMPBSA.py program of Amber12 package was used for MM-GB(PB)SA free energy calculations [48, 50] after converting the GROMACS trajectories into the Amber format by using VMD v1.9.1 as described elsewhere [51]. In all cases we followed the single trajectory (ST) approach, in which the trajectories for the free enzyme and the free ligand are extracted from that of the complex [48, 50]. GB^OBC1^, GB^OBC2^ and GB*n2* implicit-solvation models (*igb* = 2, *igb* = 5 and *igb* = 8, respectively) as well as the PB model were employed to calculate the Δ*G*_*GB/PB*_ value of each complex [29, 48, 52–54]. Topologies were obtained with tleap using mbondi2 and mbondi3 radii for GB^OBCs^ and GB*n2* models, respectively [48]. Salt concentrations of 0.50 mol/L and 0.05 mol/L were set for HNE and PPE complexes, respectively, and default solvent and solute dielectric constants (*ε*_*w*_ = 78.3 and *ε*_*in*_ = 1, respectively) and *rgbmax* cutoff (*rgbmax* = 25 Å) values were used in all GB calculations [48]. In turn, default solvent probe radius (1.4 Å), dielectric constants and grid parameters were employed to solve the PB equation [48]. Additionally, three different sets of atomic radii, i.e., mbondi2, mbondi3 and Tan and Luo pre-computed values, were utilized to calculate the solute cavity [48, 50]. In all cases, the Δ*SA* values were determined using the Linear Combination of Pair-wise Overlaps algorithm [55]. Δ*G*_*SA*_ was then estimated through Eq 1 by setting the values of *γ* and *β* to 0.0072 and 0, respectively [50]. Finally, Δ*E*_*gas*_ was estimated from AMBER99SB parameters [38]. Mean values of the energy terms were obtained by averaging over the snapshots extracted every 10 ps from each productive MD simulation. In turn, *t*_*eq*_ was estimated by the analysis of accumulated mean values of Δ*G*_*eff*_ and root mean square deviations (RMSD) during MD simulations [56]. Snapshots were considered as statistically independent from each other, since previous works have determined a typical correlation time for MM-GB(PB)SA energy terms of ~5 ps or less [57, 58].

Energetically-relevant residues, i.e., warm- and hot-spots, at the interfaces of the studied complexes were predicted by using the per-residue effective free energy decomposition (*pr*EFED) protocol implemented in MMPBSA.py [48, 50]. Of note, warm- and hot-spot residues were defined as those with a side-chain energy contribution (Δ*G*_*sc*_) to the total Δ*G*_*eff*_ value ranging from -1.0 to -0.4 kcal/mol and ≤-1.0 kcal/mol, respectively, as defined elsewhere [59]. The per-residue free energy contribution (Δ*G*_*res*_) under the ST approach is calculated as follows [28, 60]:

$$\Delta G_{res}=\frac{1}{2}\underset{i\in res,j\notin res}{\sum }\left(\Delta E_{vw}^{ij}+\Delta E_{el}^{ij}+\Delta G_{GB/PB}^{ij}\right)+\underset{i\in res}{\sum }\Delta G_{GB/PB}^{ii}+\Delta G_{SA\left(res\right)}-T\Delta S_{res}$$ (2)

The first term in the right-hand side of Eq 2 is the sum of one half of the pair-wise van der Waals ($\Delta E_{vw}^{ij}$), electrostatic ($\Delta E_{el}^{ij}$) and polar-solvation ($\Delta G_{GB/PB}^{ij}$) interaction energies between atoms *i* and *j* belonging to residue *res* and to any other residue, respectively. The second term stands for the sum of the self-interaction energies of all atoms belonging to residue *res* ($\Delta G_{GB/PB}^{ii}$). Finally, the third and fourth terms represent the per-residue non-polar solvation free energy (Δ*G*_*SA(res)*_) and entropy (*T*Δ*S*_*res*_) contributions, respectively. Δ*G*_*SA(res)*_ is calculated through the ICOSA algorithm [28, 61]; whereas *T*Δ*S*_*res*_ is neglected by default in the MMPBSA.py free energy decomposition protocol [50]. Therefore, a per-residue effective free energy rather than per-residue binding free energy decomposition was obtained.

Another approach used here to assess per-residue free energy contributions was the CAS protocol [48, 50, 60]. Briefly, Ala single-point mutations were generated at specific positions as described before and the topologies of the mutated complexes were obtained using tleap of Amber12. Subsequently, relative free energy values (ΔΔ*G*) between the native and mutated complexes were determined using MMPBSA.py. These calculations were performed under the ST approach in which the trajectory of the mutated complex is generated from that of the native complex by simply truncating the side-chain of the residue of interest and replacing the Cγ atom by a hydrogen [50, 60]. The linear correlation between the per-residue energy contributions predicted through both protocols, i.e., CAS and *pr*EFED, was assessed by estimating the Pearson coefficient (*r*_*p*_) with Mathematica v7.0 [62]. Likewise, the ability of both approaches to similarly rank the per-residue energy contributions was evaluated with the Spearman ranking coefficient (*r*_*s*_).

Finally, the pair-wise effective free energy decomposition (*pw*EFED) protocol of MMPBSA.py was employed to calculate interaction energies between pair of residues (Δ*G*_*r1*, *r2*_) [28, 48, 50]. The Δ*G*_*r1*, *r2*_ values were calculated through the following equation [28]:

$$\Delta G_{r1,r2}=\underset{\begin{array}{c} i\in r1,j\in r2,r1\neq r2\\ r1,r2\in RL \end{array}}{\sum }\left(\Delta E_{vw}^{ij}+\Delta E_{el}^{ij}+\Delta G_{GB}^{ij}\right)=\Delta E_{vw}^{r1,r2}+\Delta E_{el}^{r1,r2}+\Delta G_{GB/PB}^{r1,r2}$$ (3)

Summation in Eq 3 is carried out over all atoms *i* and *j* of residues *r1* and *r2*, respectively. Note also that the ½ factor introduced elsewhere to avoid double counting [28] has been dropped from Eq 3 in order to obtain full pair-wise interaction energies.

### Calculation of polar-desolvation and screened electrostatic energies

Some MM-GB(PB)SA energy components are associated with ideal processes which do not take place during the complex formation in solution. For example, Δ*E*_*el*_ is referred to the variation of electrostatic energy in vacuum; whereas Δ*G*_*GB/PB*_ quantifies the energy variation due to solute-solvent electrostatic interactions arisen from the transfer of individual reactant and product molecules from the vacuum to the solvent. Therefore, we used here a modification of the traditional polar-energy components (Δ*E*_*el*_ and Δ*G*_*GB*_) of the MM-GBSA method, first proposed by Zou *et al*., to assess the polar-desolvation penalty of the solute molecules (Δ*G*_*ds*_) and the screened electrostatic energy variation ($\Delta G_{el}^{sc}$) upon complex formation [63]. These energy components were calculated as follows (see S2 Fig for a derivation of Δ*G*_*ds*_ based on a thermodynamic cycle):

$$\Delta G_{ds}=\underset{i\in RL}{\sum }\Delta G_{GB}^{ii}+\underset{i,j\in R;i\neq j}{\sum }\Delta G_{GB}^{ij}+\underset{i,j\in L;i\neq j}{\sum }\Delta G_{GB}^{ij}$$ (4)

$$\Delta G_{el}^{sc}=\underset{i\in R,j\in L}{\sum }\left(\Delta E_{el}^{ij}+\Delta G_{GB}^{ij}\right)$$ (5)

It is worth saying that Eq 5 is valid only under the ST approach, since we assumed that $\sum_{i,j}\Delta E_{el}^{ij}=0$ for *i* and *j* belonging to the same molecule (either R or L).

The Δ*G*_*ds*_ and $\Delta G_{el}^{sc}$ values for the studied complexes were determined through the *pw*EFED protocol implemented in the MMPBSA.py program [48, 50]. Briefly, the output of this protocol was processed to retrieve the self (*i*, *i*)- and cross (*i*, *j*)-energy values for all residues in each complex, which were then added to obtain the total values of Δ*G*_*ds*_ and $\Delta G_{el}^{sc}$ through Eqs 4 and 5, respectively.

## Results

### Structural analysis of the protein-inhibitor interfaces during the MD simulations

#### Analysis of the interfaces of the three existing complexes

**Fig 3:**
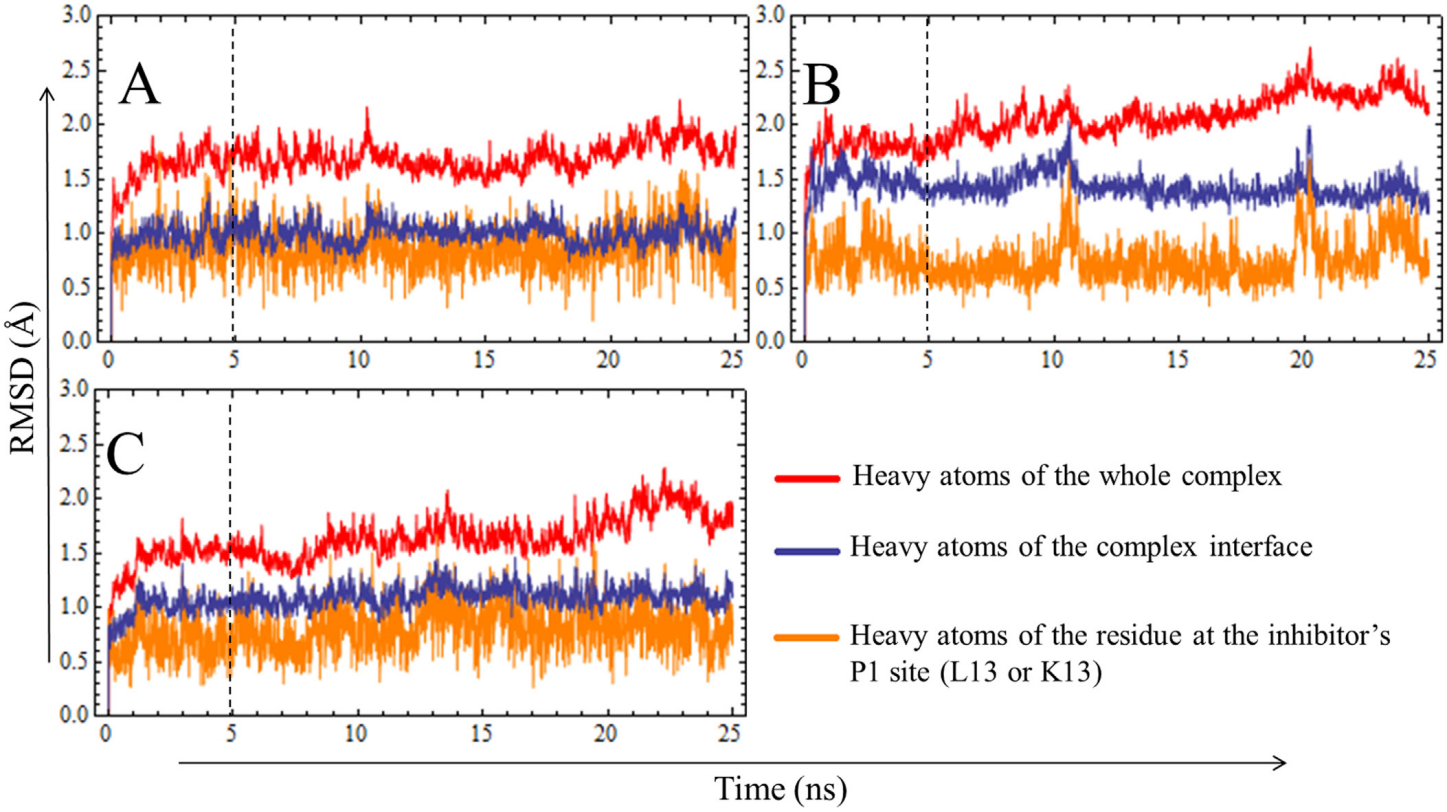
Time Evolution of Instantaneous RMSD Values for Different Heavy Atom Sets of the Three Existing Complexes. (A)HNE:ShPI-1/K13L, (B) HNE:ShPI-1, (C) PPE:ShPI-1/K13L. RMSD values with respect to the initial (*t* = 0) structure were calculated for different heavy atom sets during the MD simulation of each complex. The dashed lines represent the *t*_*eq*_ value (5 ns) chosen from the analysis of instantaneous RMSD. The complex interface was defined by using a cutoff radius of 4 Å.

The 3D models of HNE in complex with each inhibitor variant, ShPI-1 and ShPI-1/K13L, were generated by using the crystal structure of the PPE:ShPI-1/K13L complex as a suitable template (Fig 2) and were subsequently used as the starting structures for MD simulations. The stability of the three existing complexes during the MD simulations was monitored by calculating the instantaneous RMSD values for different atom sets, which were relatively stable and modularly small (<2.5 Å) after ~5 ns (= *t*_*eq*_) in all cases (Fig 3). Of note, stable RMSD time profiles were obtained for the interface heavy atoms and, particularly, for those of the inhibitor’s P1 site (Fig 3), thereby suggesting the good complementarity of the complex interfaces during the simulation time.

To validate the *in silico* structural analysis carried out here, the van der Waals contacts (≤4 Å) and hydrogen bonds occurring at the interface of the crystal structure of PPE in complex with ShPI-1/K13L were first compared to those at the corresponding representative structure obtained from the productive (*t≥t*_*eq*_) MD simulation (S1 Table). As shown in the table, most of the protease-inhibitor interactions observed in the crystal structure are also found in the representative structure of the complex. The main difference involved Q192 of PPE which underwent a side-chain rearrangement during the MD simulation, thereby forming a hydrogen bond with C12 at the P2 site of ShPI-1/K13L (S3 Fig). The overall correspondence between both structures was also inferred from the small global RMSD value (1.44 Å) calculated for their respective heavy atoms.

According to the structural analysis of the three existing complexes, most of the protease-inhibitor interactions, i.e. van der Waals contacts (S2 Table), hydrogen bonds (S3 Table) and salt bridges (S4 Table), comprised residues at positions P3-P3’ of the inhibitor’s primary binding loop and the complementary S3-S3’ subsites of both elastases. Remarkably, the residues at P3, P1, P2’ and P3’ sites of both inhibitor variants establish close van der Waals contacts with significantly-variable subsites of HNE and PPE in terms of their residue composition (S2 Table). To a lesser extent, the inhibitor’s secondary binding loop (positions P19’-P24’) is also involved in the complex formation (S2, S3 and S4 Tables).

**Fig 4:**
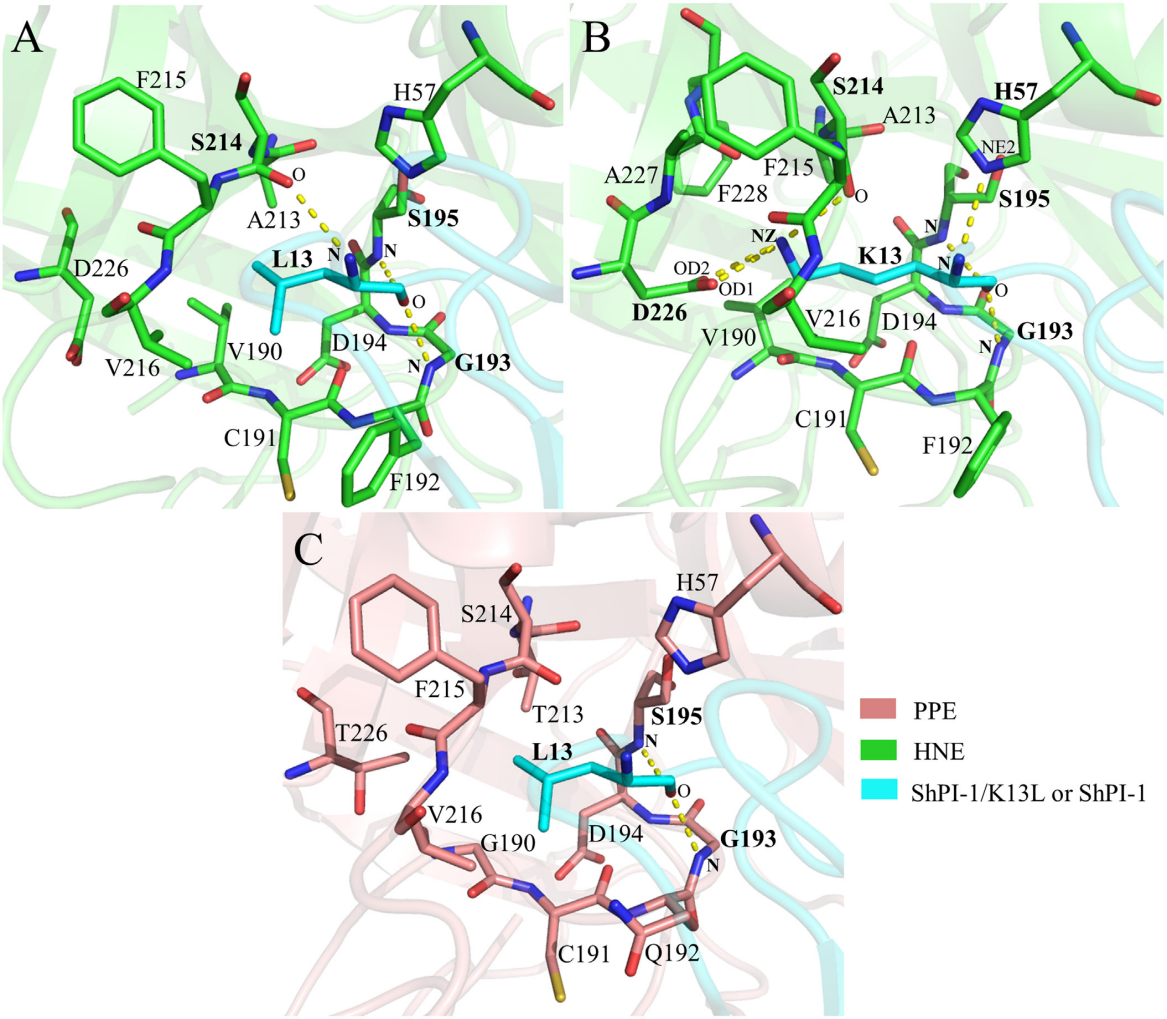
S1:P1 Interfaces of the Three Existing Complexes. (A) HNE:ShPI-1/K13L, (B) HNE:ShPI-1 and (C) PPE:ShPI-1/K13L. Hydrogen bonds with occupancies ≥40% are represented as yellow dashed lines. Residues involved in hydrogen bond formation have been labeled in bold style. Donor and acceptor atom names are labeled in bold and plain styles, respectively. Hydrogen atoms have been removed from the structures for clarity’s sake. Interfaces were defined with a cutoff radius of 4 Å. D226 has been included in (A) only for comparison purposes, since it lies at a greater distance from L13. The S1:P1 interfaces shown here correspond to the representative structures of the complexes.

As previously observed in similar complexes of canonical inhibitors and SPs [10, 13, 14], the interactions at the S1:P1 interfaces are predominant within the three protease-inhibitor interfaces analyzed here (S2 and S3 Tables). These interfaces showed both conserved and differential polar-interaction patterns among them (Fig 4). For example, the hydrogen bonds S195(**N**):P1(O) and G193(**N**):P1(O), typical of the substrate-like binding mechanism [64], were detected with high occupancies in the three complexes (Fig 4 and S3 Table). The hydrogen bonds with S214 and H57 were also found in all the interfaces, but their occupancies were rather low and variable among the complexes (S3 Table), suggesting a subordinate role of this interaction in the complex formation. The most striking difference involving the S1:P1 interfaces was detected in the HNE:ShPI-1 complex, in which two hydrogen bonds and a salt bridge are formed between D226 of HNE and K13 of ShPI-1, while similar interactions do not occur in the other two complexes (Fig 4 and S3 and S4 Tables). Hydrogen bonds also occur outside the S1:P1 interfaces of the studied complexes in agreement with previous reports for inhibitors following a substrate-like mechanism [13] (S1 Text).

Interestingly, our results also suggest that residue E44(P31’) outside the inhibitor’s primary and secondary binding loops forms a hydrogen bond and a salt bridge with R36 of HNE (S3 and S4 Tables and S5 Fig). However, in PPE the nearest positively-charged residue (R61) lies farther from E44; thereby precluding the formation of both the hydrogen bond and the salt bridge (S4 Table and S5 Fig). Note also that the side-chain of R36 in PPE extends away from the complex interface and, hence, it does not interact with E44 (S5 Fig).

#### Analysis of a hypothetical PPE:ShPI-1 interface

**Fig 5:**
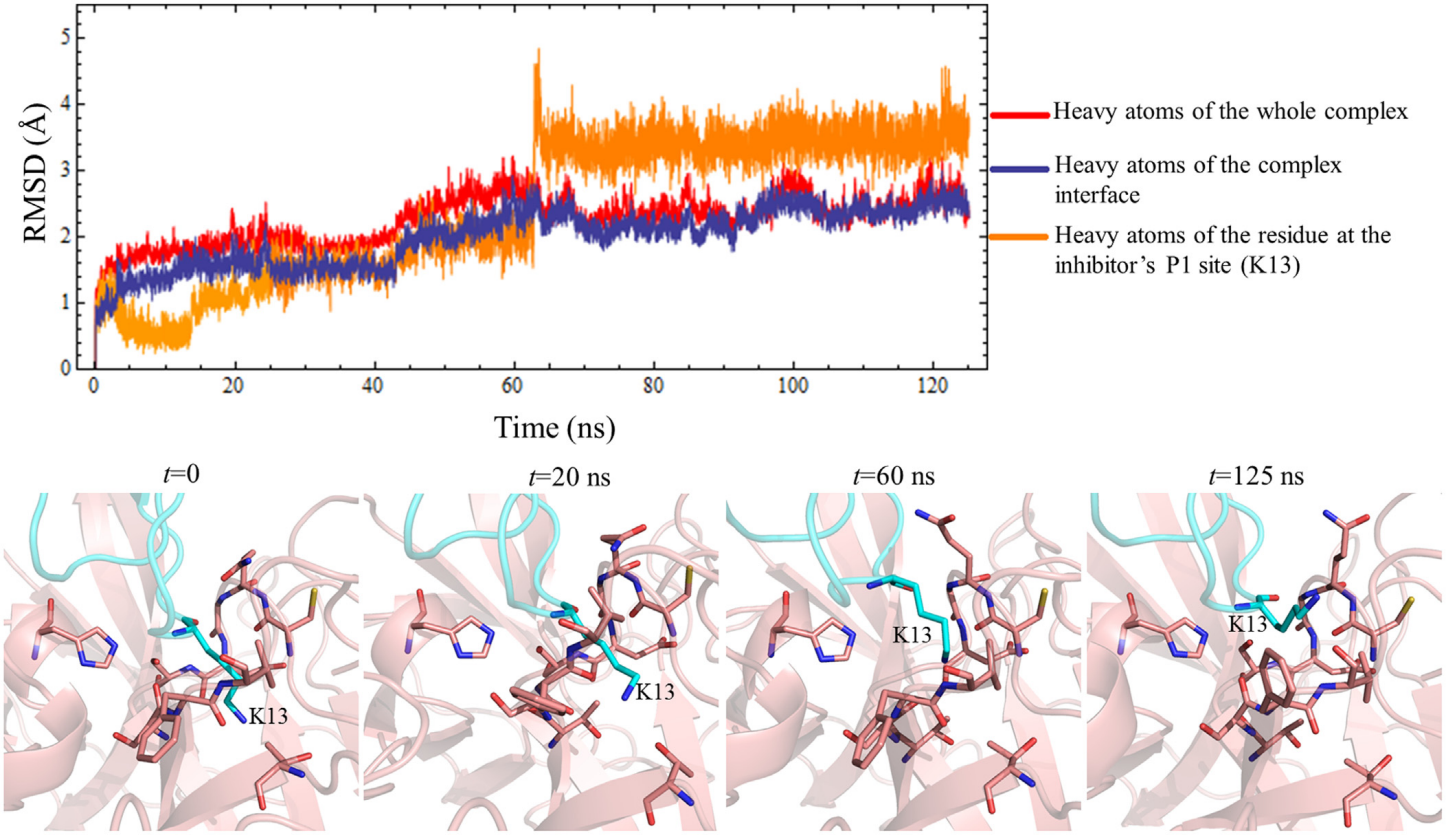
Time Profiles of RMSD Values and Structural Interface Representation for a Hypothetical PPE:ShPI-1 Complex. During the first ~43 ns a ‘in’ conformation of the PPE:ShPI-1 complex in which the side-chain of K13 lies within the S1 subsite of PPE was sampled (see structures at *t* = 0 and *t* = 20 ns). From *t*≈43 ns to *t*≈62 ns, a reorganization of the complex interface occurred in order to provide space for K13 exit (see structure at *t* = 60 ns). Finally, at *t*≈62 ns the side-chain of K13 changed to an ‘up’ conformation and remained in that conformation up to 125 ns (see structure at *t* = 125 ns).

The starting structure of a hypothetical PPE:ShPI-1 complex was subjected to a 125 ns MD simulation. The time profiles of RMSD values calculated for distinct sets of protein heavy atoms indicate the occurrence of conformational changes at the complex interface during the MD simulation (Fig 5). As revealed by visual inspection of different frames, these conformational changes comprised the complex interface reorganization leading to the further exit of the K13 side-chain from the S1 subsite. In fact, the side-chain of this residue sampled two main conformations during the MD simulation *i*) an ‘in’ conformation inside the S1 subsite of PPE and *ii*) an ‘up’ conformation at the entrance of this subsite. It is worth noting that the nomenclature PPE:ShPI-1*in* and PPE:ShPI-1*up* will be used hereinafter to refer to the whole complexes bearing the ‘in’ and ‘up’ conformations of K13 side-chain, respectively. As shown in Fig 5 the ‘in conformation’ of K13 transiently exited the S1 subsite until the ‘up’ conformation was reached at *t≈*62 ns, but no further evidence of instability of the complex interface was observed up to 125 ns. Notwithstanding, the occurrence of complete interface disruption at longer simulation times cannot be discarded.

The analysis of interactions similar to that performed for the three existing complexes was carried out for a hypothetical PPE:ShPI-1 complex. The PPE:ShPI-1*in* and PPE:ShPI-1*up* complexes were analyzed independently from two different time intervals of the MD simulation, each sampling one of the K13 side-chain conformations (from 5 ns to 25 ns, and from 70 ns to 90 ns, respectively). The van der Waals contacts across the PPE:ShPI-1*in* interface were similar to those at the PPE:ShPI-1/K13L interface (S2 and S5 Tables). However, the PPE:ShPI-1*up* complex displayed a remarkable reduction of van der Waals contacts at the Sn:Pn (n = 1, 2, 3, 4 and 5) interfaces, whereas no noticeable differences were observed at the Sn’:Pn’ interfaces of both conformations (S5 Table).

**Fig 6:**
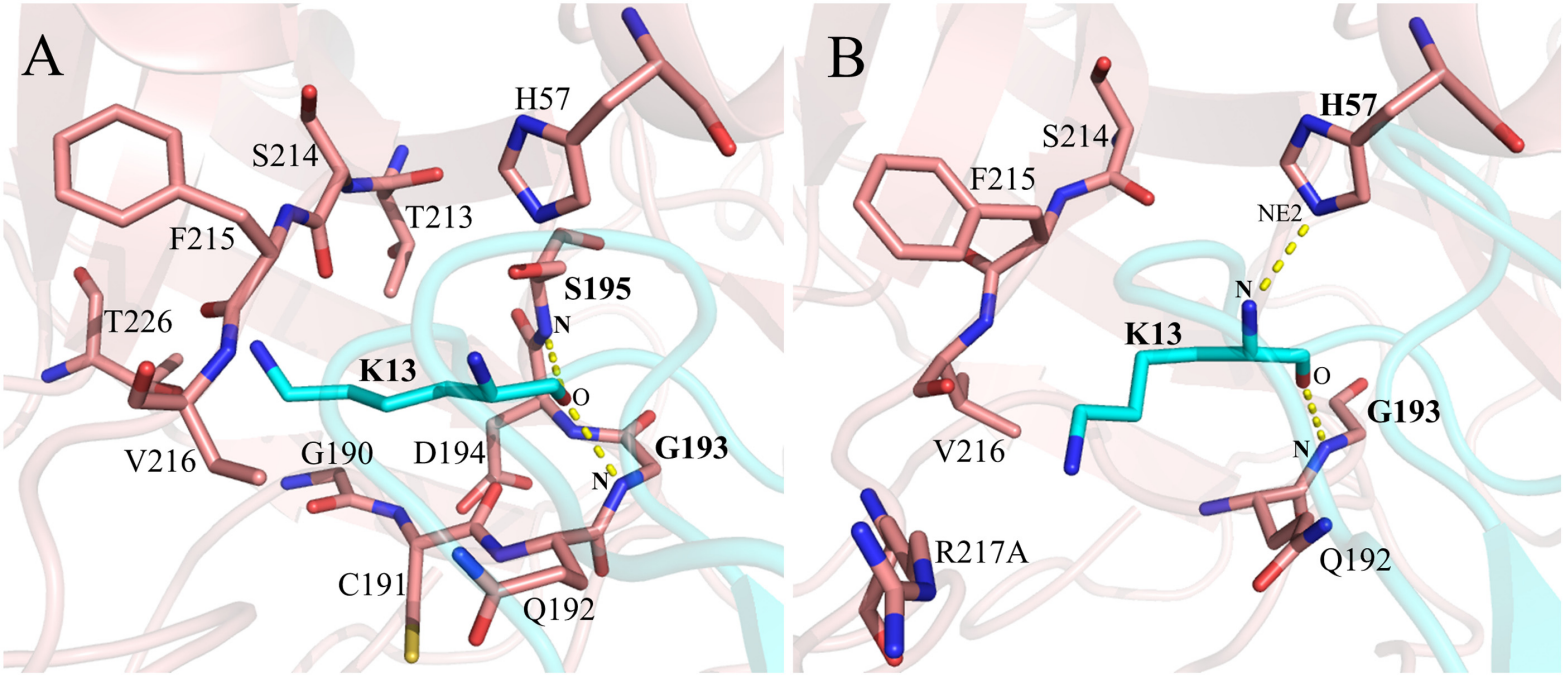
S1:P1 Interfaces of the Two Alternative Conformations of the PPE:ShPI-1 Complex Sampled during the MD Simulation. (A) PPE:ShPI-1*in* and (B) PPE:ShPI-1*up* complex. Hydrogen bonds with occupancies ≥40% are represented as yellow dashed lines. Residues involved in hydrogen bond formation have been labeled in bold. Donor and acceptor atom names are labeled in bold and plain styles, respectively. Hydrogen atoms have been removed from the structures for clarity’s sake. The S1:P1 interfaces shown here correspond to the representative structures of the complexes.

The hydrogen bond and salt bridge patterns at the PPE:ShPI-1*in* interface are similar to those of the PPE:ShPI-1/K13L complex (S3, S4 and S6 Tables). Remarkably, the K13 side-chain did not establish stable polar interactions within the S1 subsite of PPE (Fig 6 and S6 Table). Indeed, T226 of PPE only forms a very weak hydrogen bond with the ε-amine group of K13, whereas D226 of HNE forms stable hydrogen bonds and a salt bridge with the latter group, as previously stated (Figs 4 and 6 and S3, S4 and S6 Tables). On the other hand, the PPE:ShPI-1*up* interface possesses a reduced number of hydrogen bonds compared to that of the PPE:ShPI-1*in* complex, especially, at the S3:P3 and S2:P2 interfaces. It is worth noting that the hydrogen bond S195(**N**):K13(O), conserved throughout the other interfaces analyzed here, and characteristic of the substrate-like interaction mechanism, is abrogated in the ‘up’ conformation (Fig 6B). Overall, a decrease in both the van der Waals and polar interactions at the complex interface, mainly involving the Pn side of the inhibitor binding loop and the complementary enzyme subsites, was observed upon the transition from the ‘in’ to the ‘up’ conformation. In addition, the ε-amine group of K13 is not stabilized through polar interactions with the PPE residues in any conformation.

#### Different outcomes upon small disruption of the interfaces of ShPI-1 in complex with PPE and HNE

The MD simulation of the PPE:ShPI-1 complex showed the exit of the K13 side-chain from the S1 subsite of the enzyme; however, an eventual dissociation of the complex was not sample up to 125 ns (Fig 5). Instead of conducting longer simulations, we decided to assess whether ShPI-1 remains bound to the enzyme upon an artificial disruption of the complex interface. The same exact protocol was carried out for the HNE:ShPI-1 complex, which is expected to remain associated upon slight disruption (positive control).

**Fig 7:**
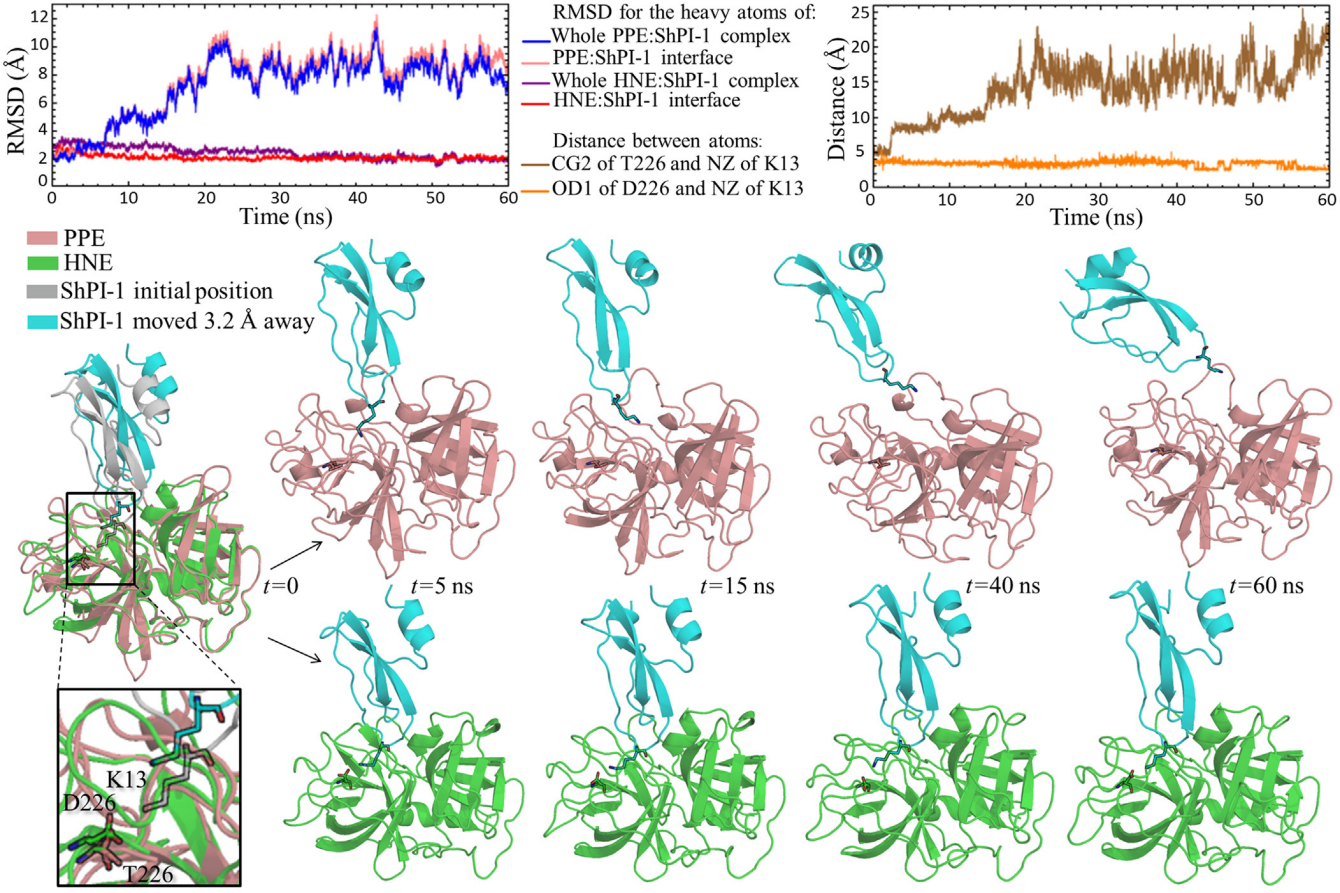
Different Outcomes of the MD Simulations Performed for the PPE:ShPI-1 and HNE:ShPI-1 Complexes upon Slight Interface Disruption. The time profiles of RMSD values calculated for different atom sets as well as the distance between T226(CG2)/D226(OD1) of PPE/HNE and K13(NZ) of ShPI-1 as a function of time are shown for each complex. The structures of some frames extracted from each MD simulation are also depicted. Note that the disrupted starting structures of each complex (*t* = 0) were superimposed prior to the representation and that the initial position of ShPI-1 (gray) is shown only for comparison purposes. K13 of ShPI-1 and D226/T226 of HNE/PPE are depicted in stick representation.

The outcome of this experiment is presented in Fig 7. As can be seen, ShPI-1 readily dissociates from PPE during the 60 ns MD simulation of the disrupted complex. This suggests, in turn, that the association of ShPI-1 to PPE is an unfavorable process, since a nearly-effective collision did not lead to complex formation. In contrast, ShPI-1 remained bound to HNE upon disruption (Fig 7), thereby suggesting the stability of this complex. Hence, ShPI-1 shows a differential ability to interact with PPE and HNE, in agreement with the experimental data [7]. In addition, the distance between the T226(CG2)/D226(OD1) atom of PPE/HNE and the K13(NZ) atom of ShPI-1 as a function of time was calculated (Fig 7). As can be observed, the distance between K13(NZ) and D226(OD1) decreases during the MD simulation until it reaches a plateau at ~2.7 Å, indicating the propensity of these oppositely-charged groups to restore the sat bridge interaction after the interface disruption. Conversely, the distance between T226(CG2) and K13(NZ) increases during the MD simulation, consistently with their inability to establish strong pair-wise interactions (S6 Table). Hence, we propose that the formation of the PPE:ShPI-1 complex may be impaired by an unfavorable association process, disregarding the possibility of a slow dissociation rate of the complex from hypothetical bound states. Moreover, the salt bridge between D226 of HNE and K13 of ShPI-1 may be essential to the complex formation, whereas T226 of PPE seems to be irrelevant for ShPI-1 binding.

### Effective free energy calculations and correlation with experimental data

The large difference in the affinity of ShPI-1 and ShPI-1/K13L for PPE cannot be straightforwardly deduced from the differential interaction patterns at the interfaces of both complexes (Figs 4C and 6, and S2, S3, S4, S5 and S6 Tables). Furthermore, the HNE:ShPI-1 complex has more favorable overall interactions than the other three (Fig 4A and 4B, S2, S3 and S4 Tables), but its *K*_*i*_ value (2.35·10^−8^ M) is higher than those of the HNE:ShPI-1/K13L (1.3·10^−9^ M) and the PPE:ShPI-1/K13L complex (1.2·10^−8^ M) [7]. These discrepancies demonstrate that, in principle, it is not possible to establish a qualitative relation between the number and/or nature of the interface contacts and the binding affinity of the complexes, as has been pointed out elsewhere [65, 66]. Therefore, free energy calculations are needed to determine the energetic factors underlying the relative affinity values of these systems.

**Fig 8:**
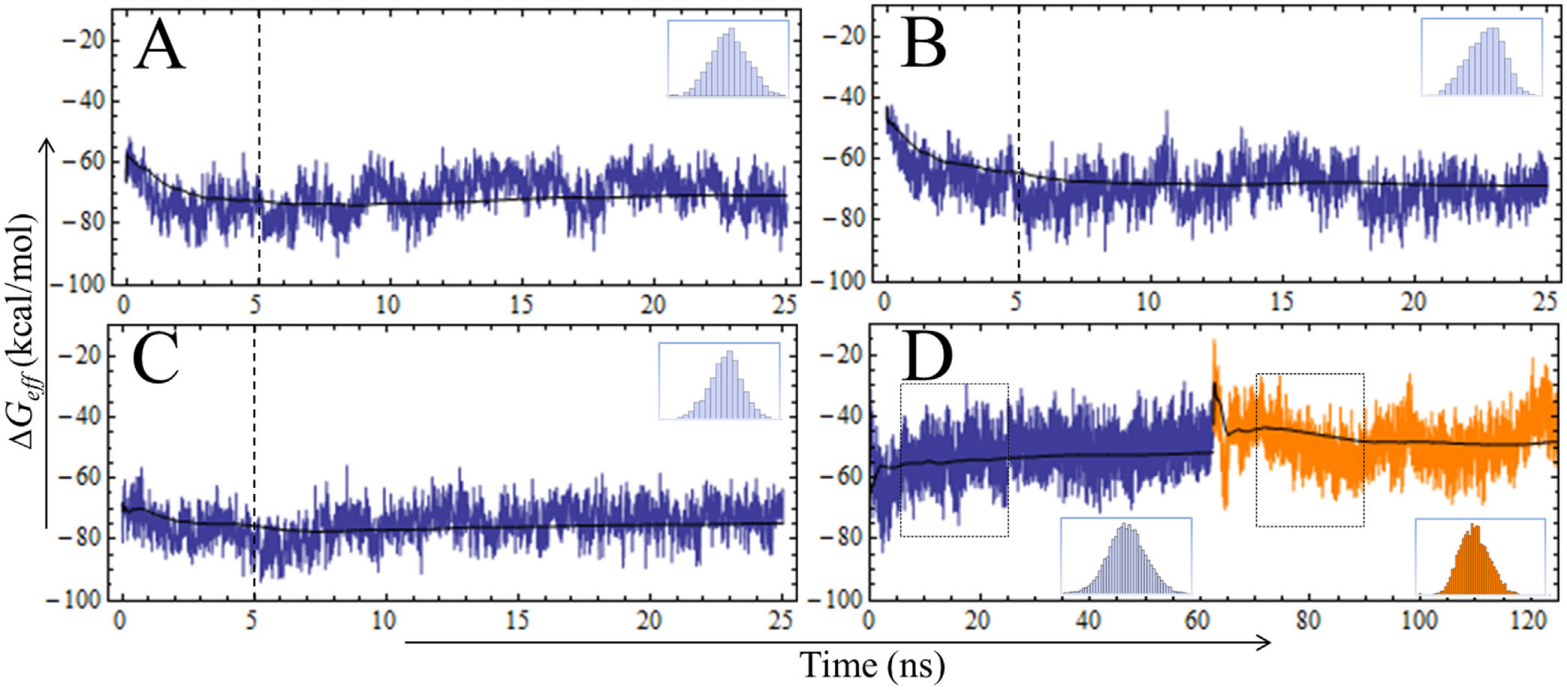
Time Evolution of Instantaneous Δ*G*_*eff*_ Values for the Four Complexes. (A) HNE:ShPI-1/K13L, (B) HNE:ShPI-1, (C) PPE:ShPI-1/K13L and (D) PPE:ShPI-1. Instantaneous Δ*G*_*eff*_ values (in blue) were calculated using the GB^OBC2^ model (*igb* = 5). Histogram insets indicate the normal distribution of the Δ*G*_*eff*_ values. The Δ*G*_*eff*_ values corresponding to the PPE:ShPI-1*in* and PPE:ShPI-1*up* complexes are depicted in blue and orange, respectively. The black line indicates the accumulated mean value of Δ*G*_*eff*_ for each trajectory. The dashed line represents the *t*_*eq*_ value (5 ns) determined from the analysis of instantaneous RMSD and Δ*G*_*eff*_ values. Frames selected for Δ*G*_*eff*_ calculations in (D) are indicated by dashed rectangles.

According to the instantaneous Δ*G*_*eff*_ values calculated during the MD simulations of the three existing complexes, relatively stable accumulated mean values of Δ*G*_*eff*_ were reached after the first 5 ns (Fig 8A, 8B and 8C). Since a similar behavior was observed in the RMSD time series (Fig 3), we considered 5 ns as a suitable *t*_*eq*_ value. Therefore, the Δ*G*_*eff*_ mean values were calculated using the last 20 ns of each productive MD simulation. Note, however, that an abrupt instability in the time profile of the PPE:ShPI-1 complex was observed at *t≈*62 ns, which corresponds to the exit of K13 side-chain from the S1 subsite (compare Figs 5 and 8D). Hence, for this particular complex, the free energy calculations were carried out independently for the same two MD simulation intervals employed before in the structural analysis.

The Δ*G*_*eff*_ mean values for each complex, estimated through different GB and PB models, are shown in S7 Table; whereas the calculated relative free energy values (ΔΔ*G*_*calc*_) and *K*_*i*_ ratios for all complexes are shown in Table 1. In particular, the comparison of the calculated (ΔΔ*G*_*calc*_) and experimental (ΔΔ*G*_*exp*_) relative free energy values for the interaction of both inhibitor variants with a given elastase, revealed that all the implicit-solvation models consistently predicted the higher affinity of ShPI-1/K13L for PPE, whereas only the GB^OBC2^ model predicted the higher affinity of this inhibitor variant for HNE (Table 1A). In fact, the ΔΔ*G*_*calc*_ value for the HNE complexes obtained with this GB model is only 0.92 kcal/mol lower than the experimental value, which is an acceptable estimation error, especially when considering that the relative entropy contribution (*T*·ΔΔ*S*) was excluded from the ΔΔ*G* prediction. According to the ΔΔ*G*_*calc*_ value for the PPE complexes obtained through the GB^OBC2^ model (Table 1A), a *K*_*i*_ value of 2.18·10^8^ M^-1^ for the binding of ShPI-1 to PPE is obtained from the experimental *K*_*i*_ value of the PPE:ShPI-1/K13L complex and the ΔΔ*G*_*calc*_ value. Hence, based on the previous *K*_*i*_ value, there must be not complex formation under the experimental conditions employed in the inhibition assays, which agrees with the reported data [7].

**Table 1:** Calculated and Experimental ΔΔ*G* Values and *K*_*i*_ Ratios.

**(A)**				
**Enzyme**	**ΔΔ*G***_***calc***_ ^**a**^ **(kcal/mol)**	**ΔΔ*G***_***exp***_ ^**b**^ **(kcal/mol)**	$K_{i,calc}^{\left(1\right)}/K_{i,calc}^{\left(2\right)}$ ^**c**^	$K_{i,exp}^{\left(1\right)}/K_{i,exp}^{\left(2\right)}$ ^**d**^
PPE^e^	-19.64±0.21 (2)^f^	-^h^	(3.94±1.44)·10^−15^ (2)	-^h^
	-22.17±0.23 (5)^f^		(5.49±2.21)·10^−17^ (5)	
	-17.67±0.19 (8)^f^		(1.09±0.36)·10^−13^ (8)	
	-14.07±0.35 (pb1)^g^		(4.79±2.89)∙10^−11^ (pb1)	
	-19.91±0.26 (pb2)^g^		(2.49±1.13)∙10^−15^ (pb2)	
	-20.65±0.25 (pb3)^g^		(7.16±3.12)∙10^−16^ (pb3)	
HNE	3.80±0.18 (2)	-1.71±0.23	(6.09±0.18)·10^2^ (2)	(5.53±2.17)·10^−2^
	-0.79±0.20 (5)		(2.63±0.89)·10^−1^ (5)	
	6.09±0.15 (8)		(2.92±0.73)·10^4^ (8)	
	20.53±0.28 (pb1)		(1.14±0.52)·10^15^ (pb1)	
	6.35±0.22 (pb2)		(4.54±1.67)·10^4^ (pb2)	
	3.49 ± 0.19 (pb3)		(3.62±1.15)·10^2^ (pb3)	
**(B)**				
**Inhibitor**	**ΔΔ*G***_***calc***_ **(kcal/mol)**	**ΔΔ*G***_***exp***_ **(kcal/mol)**	**$K_{i,calc}^{\left(1\right)}/K_{i,calc}^{\left(2\right)}$**	**$K_{i,exp}^{\left(1\right)}/K_{i,exp}^{\left(2\right)}$**
ShPI-1	-15.49±0.21 (2)	-	(4.35±1.58)·10^−12^ (2)	-
	-17.40±0.23 (5)		(1.73±0.69)·10^−13^ (5)	
	-16.69±0.19 (8)		(5.74±1.89)·10^−13^ (8)	
	-28.12±0.36 (pb1)		(2.37±1.51)∙10^−21^ (pb1)	
	-17.79±0.28 (pb2)		(8.96±4.35)∙10^−14^ (pb2)	
	-14.14±0.27 (pb3)		(4.26±1.98)∙10^−11^ (pb3)	
ShPI-1/K13L	7.95±0.18 (2)	-1.32±0.31	(6.78±2.03)·10^5^ (2)	(1.08±0.56)·10^−1^
	3.98±0.20 (5)		(8.30±2.78)·10^2^ (5)	
	7.07±0.16 (8)		(1.78±0.41)·10^5^ (8)	
	6.47±0.25 (pb1)		(5.50±2.32)∙10^4^ (pb1)	
	8.47±0.20 (pb2)		(1.62±0.54)∙10^6^ (pb2)	
	10.00±0.18 (pb3)		(2.15±0.64)∙10^7^ (pb3)	

^a^(A) ΔΔ*G*_*calc*_ = Δ*G*_*eff*_(E:ShPI-1/K13L)-Δ*G*_*eff*_(E:ShPI-1), where E stands for either PPE or HNE, (B) ΔΔ*G*_*calc*_ = Δ*G*_*eff*_(HNE:I)-Δ*G*_*eff*_(PPE:I), where I stands for either ShPI-1 or ShPI-1/K13L.

^b^(A) ΔΔ*G*_*exp*_ = *RTlnK*_*i*_(HNE:ShPI-1/K13L)/*K*_*i*_(HNE:ShPI-1), or (B) ΔΔ*G*_*exp*_ = *RTlnK*_*i*_(HNE:ShPI-1/K13L)/*K*_*i*_(PPE:ShPI-1/K13L), where the *K*_*i*_ values were experimentally determined.

^c^(A) $K_{i,calc}^{\left(1\right)}/K_{i,calc}^{\left(2\right)}=K_{i,calc}^{\left(E:ShPI-1\right)}/K_{i,calc}^{\left(E:ShPI-1/K13L\right)}=\text{exp}\left(\Delta \Delta G/RT\right)$, where E stands for either PPE or HNE, (B) **$K_{i,calc}^{\left(1\right)}/K_{i,calc}^{\left(2\right)}=K_{i,calc}^{\left(ENH:I\right)}/K_{i,calc}^{\left(EPP:I\right)}=\text{exp}\left(\Delta \Delta G/RT\right)$**, where I stands for either ShPI-1 or ShPI-1/K13L.

^d^Experimental *K*_*i*_ values taken from Garcia-Fernandez *et al* [7].

^e^For ΔΔ*G* calculations an average Δ*G*_*eff*_ value of PPE:ShPI-1 complex obtained from those of the two conformations (S7 Table) was employed.

^f^The GB model used for ΔΔ*G* calculation is indicated by the value of the *igb* variable between parentheses.

^g^The PB model used for for ΔΔ*G* calculation is indicated between parentheses, where pb1, pb2 and pb3 stand for the PB model using Tan and Luo, mbondi2 and mbondi3 atomic radii, respectively.

^h^No PPE:ShPI-1 complex formation has been experimentally detected, hence, ΔΔ*G*_*exp*_ value is expected to be a large and negative number, and the *K*_*i*_ ratio must tend to zero.

Additionally, the ΔΔ*G*_*calc*_ values and *K*_*i*_ ratios for the binding of each inhibitor variant to both elastases were estimated using the corresponding Δ*G*_*eff*_ values (Table 1B). As can be observed from the table, all implicit-solvation models correctly predicted the higher affinity of ShPI-1 for HNE. However, none of the GB models was able to correctly rank the affinities of ShPI-1/K13L for both elastases (Table 1B). Apart from the inaccuracies of the implicit-solvation models, the previous result may be influenced by the exclusion of the *T∙*ΔΔ*S* term from the ΔΔ*G*_*calc*_ calculations, an invalid assumption for systems bearing large structural differences [48]. In fact, in contrast to the ΔΔ*G*_*calc*_ values shown in Table 1A, the systems compared in Table 1B comprise two distinct enzymes, which, in turn, possess several point variations in the residue composition of their binding sites (see S1 Fig for a comparison of the S1 subsites of PPE and HNE). In spite of that, the ΔΔ*G*_*calc*_ value for ShPI-1/K13L in complex with each enzyme predicted by the GB^OBC2^ model is again in better agreement with the corresponding ΔΔ*G*_*exp*_ value than those obtained with the GB^OBC1^, GB*n2* and the three PB variants (Table 1B). Interestingly, a *T∙*ΔΔ*S* value of 5.30 kcal/mol would explain the difference between the ΔΔ*G*_*calc*_ value obtained with the GB^OBC2^ model and the ΔΔ*G*_*exp*_ value for the previous complexes. In turn, the same *T∙*ΔΔ*S* value would not qualitatively affect the prediction of the relative affinity of ShPI-1 for both enzymes, i.e., a large relative affinity would be obtained, ΔΔ*G*_*calc*_ = -22.70 kcal/mol.

Of note, the ability of GB models to yield better predictions than the PB model, in theory more accurate, is not a surprising result in light of previous works [67–70]. Since the GB^OBC2^ model accounted for the experimental relative affinities of the PPE and HNE complexes, this model was used in all further energy calculations, without an explicit mention of it hereinafter.

### The effect of desolvation and protein-protein interactions on the formation of the studied complexes in solution

Polar-desolvation and screened electrostatic energies associated with the formation of the complexes in solution, in addition to the van der Waals and non-polar solvation energies, are shown in Table 2. These results show that ShPI-1 establishes stronger screened electrostatic interactions with HNE than ShPI-1/K13L ($\Delta \Delta G_{el}^{sc}$ = 37.81 kcal/mol, Table 2). A similar behavior—although less pronounced—was obtained for the van der Waals and non-polar solvation energies (Table 2). However, the polar-desolvation penalty for the binding of ShPI-1 to HNE is larger than that of ShPI-1/K13L (ΔΔ*G*_*ds*_ = -42.63 kcal/mol, Table 2). This fact determines the more favorable Δ*G*_*eff*_ value of the HNE:ShPI-1/K13L complex.

**Table 2:** Polar and non-Polar MM-GBSA Energy Components Associated with the Complex Formation in Solution.

Complex	$\Delta G_{el}^{sc}$ ^a^ (kcal/mol)	Δ*G*_*ds*_ ^a^ (kcal/mol)	Δ*E*_*vw*_ ^b^ (kcal/mol)	Δ*G*_*SA*_ ^b^ (kcal/mol)
HNE:ShPI-1/K13L	-53.54±0.18^c^	83.28±0.45	-90.13±0.09	-10.14±0.01
HNE:ShPI-1	-91.35±0.24	125.91±0.53	-92.72±0.11	-11.55±0.01
***ΔΔE*** ^**d**^ **(kcal/mol)**	37.81±0.30	-42.63±0.69	2.59±0.14	1.41±0.01
PPE:ShPI-1/K13L	-60.42±0.17	106.59±0.51	-108.62±0.10	-12.31±0.01
PPE:ShPI-1*in*	-68.30±0.26	122.18±0.60	-96.64±0.14	-11.55±0.02
PPE:ShPI-1*up*	-41.34±0.23	96.65±0.57	-94.56±0.12	-11.10±0.01
***ΔΔE***_***in***_ ^**e**^ **(kcal/mol)**	7.88±0.31	-15.59±0.78	-11.98±0.17	-0.76±0.02
***ΔΔE***_***up***_ ^**e**^ **(kcal/mol)**	-19.08±0.28	9.94±0.76	-14.06±0.16	-1.21±0.02

^a^Polar energy components.

^b^Non-polar energy components.

^c^Mean value ±standard deviation of the mean.

^d^ΔΔ*E* stands for the difference between the energy component values of the same column.

^e^ΔΔ*E*_*in*_ = Δ*E*(PPE:ShPI-1/K13L)- Δ*E*(PPE:ShPI-1*in*) and ΔΔ*E*_*up*_ = Δ*E*(PPE:ShPI-1/K13L)-Δ*E*(PPE:ShPI-1*up*)

For the PPE complexes, a larger van der Waals energy contribution for the ShPI-1/K13L binding was predicted with respect to the ‘in’ conformation of the wild-type inhibitor (ΔΔ*E*_*vw*_ = -11.98 kcal/mol, Table 2). Conversely, the screened electrostatic interactions of the PPE:ShPI-1*in* complex are more favorable ($\Delta \Delta G_{el}^{sc}$ = 7.88 kcal/mol, Table 2), but the global pair-wise interaction energy is still larger for the PPE:ShPI-1/K13L complex ($\Delta \Delta G_{el}^{sc}+\Delta \Delta E_{vw}=-4.10$ kcal/mol). Note, however, that the relative contribution of pair-wise interactions does not account for the large ΔΔ*G*_*calc*_ value between these complexes, which mainly arises from their relative polar-desolvation penalty (ΔΔ*G*_*ds*_ = -15.59 kcal/mol, Table 2). On the other hand, there is a significant reduction of the polar-desolvation penalty in the PPE:ShP-1*up* complex with respect to both the PPE:ShPI-1*in* an the PPE:ShPI-1/K13L complex (Table 2). Nonetheless, the van der Waals interactions and, more importantly, the screened electrostatic interactions are remarkably less favorable in the former complex (Table 2). Furthermore, the Δ*G*_*SA*_ value is less favorable for the PPE:ShP-1*up* complex than for the other two PPE complexes (Table 2), indicating a decrease in the interface surface of the former. Overall, the weaker relative affinity of ShPI-1 for PPE is caused by the higher polar-desolvation penalty associated with the formation of the PPE:ShPI-1*in* complex, in addition to the less favorable pair-wise interactions at its interface when compared to those of the PPE:ShPI-1/K13L complex. An alternative ‘up’ conformation of the former complex is neither favorable due to the weak pair-wise interactions associated with its formation.

#### The impact of the K13L mutation on the polar-desolvation penalty

**Fig 9:**
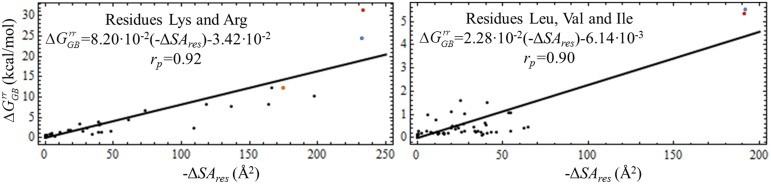
Linear Correlation between the Variation of Per-Residue Self-Energies and Solvent Accessible Surface Areas upon Complex Formation. The variation of per-residue self-energies ($\Delta G_{GB}^{rr}$) for the selected residues was obtained from the outputs of the *pw*EFED protocol, and correlated with corresponding opposite values of per-residue solvent-accessible surface area variation (-Δ*SA*_*res*_) In turn, Δ*SA*_*res*_ values were calculated from the Δ*G*_*SA(res)*_ values present in the output of the *pr*EFED protocol through the following equation Δ*SA*_*res*_ = Δ*G*_*SA(res)*_/0.0072 (see Eq 1). The blue, orange and red dots on the left-hand panel correspond to K13 in the PPE:ShPI-1*in*, PPE:ShPI-1*up* and HNE:ShPI-1 complexes, respectively. On the right-hand panel, the red and blue dots correspond to L13 of ShPI-1/K13L in complex with HNE and PPE, respectively. The N-terminal residues I16 and V16 of HNE and PPE, respectively, were ruled out from the analysis, since the presence of a positively-charged amine group changes the overall hydrophobicity of these residues. The Pearson correlation coefficient for each linear fit is represented by *r*_*p*_. The linear-fit slopes represent an inverse measurement of the average hydrophobicity of each group of similar residues, i.e., the higher the slope value the lesser the residue hydrophobicity.

The formation of the PPE:ShPI-1*in* and the HNE:ShPI-1 complex occurs at expense of larger polar-desolvation penalties compared to those of both ShPI-1/K13L complexes (Table 2). As observed, the greater affinity of the mutated variant for these enzymes is mostly determined by the ΔΔ*G*_*ds*_ values (Table 2). To better understand the impact of the K13L mutation on the ΔΔ*G*_*ds*_ values, we performed a linear correlation analysis between the per-residue self-energy ($\Delta G_{GB}^{rr}$) and Δ*SA*_*res*_ values for all positively-charged (Lys and Arg) and non-polar aliphatic (Leu, Ile, Val) residues in the studied complexes (Fig 9). According to our predictions, the molar energy required to desolvate a surface area of 1 Å^2^ of positively-charged residues (8.20∙10^−2^ kcal∙Å^-2^∙mol^-1^) is ~3.60 times higher than that required for the non-polar aliphatic residues (2.28∙10^−2^ kcal∙Å^-2^∙mol^-1^) (Fig 9). Thus, we inferred that the large $\Delta \Delta G_{GB}^{rr}$ values (and, consequently, the ΔΔ*G*_*ds*_ values) associated with the K13L mutation are caused by the hydrophobicity differences between the Lys and Leu residues, together with the large desolvation underwent by both P1 residues upon their insertion into the S1 subsite, i.e., │Δ*SA*_*K13*_ *│*~230 Å^2^ and *│*Δ*SA*_*L13*_ *│*~190 Å^2^, respectively (Fig 9).

Moreover, the results presented here indicate that the polar-desolvation penalty of K13 dramatically decreases in the ‘up’ conformation when compared to the ‘in’ conformation of the PPE:ShPI-1 complex, due to the reduction of the desolvation of this residue in the former conformation (Fig 9). Additionally, other residues at the PPE:ShPI-1*up* interface displayed lower desolvation penalties, especially those lying at the Pn and Sn sides of the inhibitor and the enzyme, respectively (data not shown). Note, however, that the polar-desolvation decrease is concomitant to a reduction of the PPE:ShPI-1*up* pair-wise interaction energy (Table 2), which ultimately determines the low affinity of this complex.

#### Energy contributions of residues at the complex interfaces

The identification of warm-/hot-spots at the interfaces of the studied complexes was first carried out using the *pr*EFED protocol (S8, S9 and S10 Tables). This method predicted a relative free energy contribution of the P1 site residues in both HNE complexes significantly different from the ΔΔ*G*_*calc*_ value of the whole complexes (ΔΔ*G*_sc(L13-K13)_ = -7.52 kcal/mol and ΔΔ*G*_*calc*_ = -0.79 kcal/mol, see S8 Table and Table 1A, respectively). Thus, we performed the CAS protocol for all the residues previously identified as warm/hot-spots by the *pr*EFED approach. Of note, we excluded from this procedure the Cys, Pro, Gly and Ala residues, for which the mutation by Ala is either structurally forbidden or meaningless.

**Fig 10:**
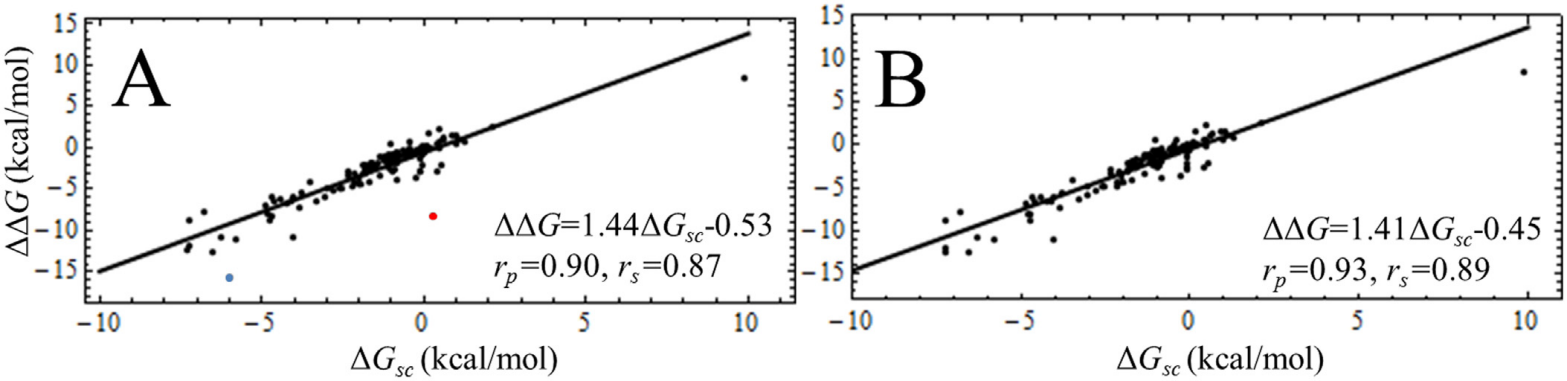
Linear Correlation between the *pr*EFED (Δ*G*_*sc*_) and CAS (ΔΔ*G*) Results for Residues of the Studied Complexes. (A) All the selected residues (S8, S9 and S10 Tables) were considered in the correlation analysis, including D226 and K13 of the HNE:ShPI-1 complex (blue and red dots, respectively). (B) Same as in (A) but excluding residues D226 and K13. The linear-fit equations and the Pearson and Spearman coefficients (*r*_*p*_ and *r*_*s*_, respectively) are shown.

The predictions of CAS and *pr*EFED were linearly correlated (*r*_*p*_ = 0.90) and similarly ranked the residues according to their energy contributions (*r*_*s*_ = 0.87) (Fig 10A), in accordance with previous reports [60]. As observed, the points corresponding to D226 and K13 of the HNE:ShPI-1 complex are outliers of the linear fit, since their elimination from the statistical analysis improved the values of the correlation coefficients (Fig 10B). This fact proved that the energy predictions of CAS and *pr*EFED significantly differed for both residues. Moreover, only CAS yielded a relative free energy contribution for K13 and L13 at the P1 site of both inhibitors consistent with the ΔΔ*G*_*calc*_ value (ΔΔ*G*_*(L13-K13)*_ = -0.46 kcal/mol). This discrepancy is likely to be caused by the well-known proneness of GB models to errors in the calculation of individual $\Delta G_{GB}^{ij}$ values, which nearly cancel during the calculation of total values ($\sum_{i,j}\Delta G_{GB}^{ij}$) [71–73]. Since CAS predictions are based on the Δ*G*_*eff*_ values for the native and the mutated complex, it becomes apparent that they are less influenced by internal errors in the $\Delta G_{GB}^{ij}$ calculations than those obtained through *pr*EFED. Overall, we concluded that CAS was more robust than *pr*EFED, especially when predicting the energy contributions of residues involved in salt-bridge interactions, like D226 and K13 of the HNE:ShPI-1 complex.

**Fig 11:**
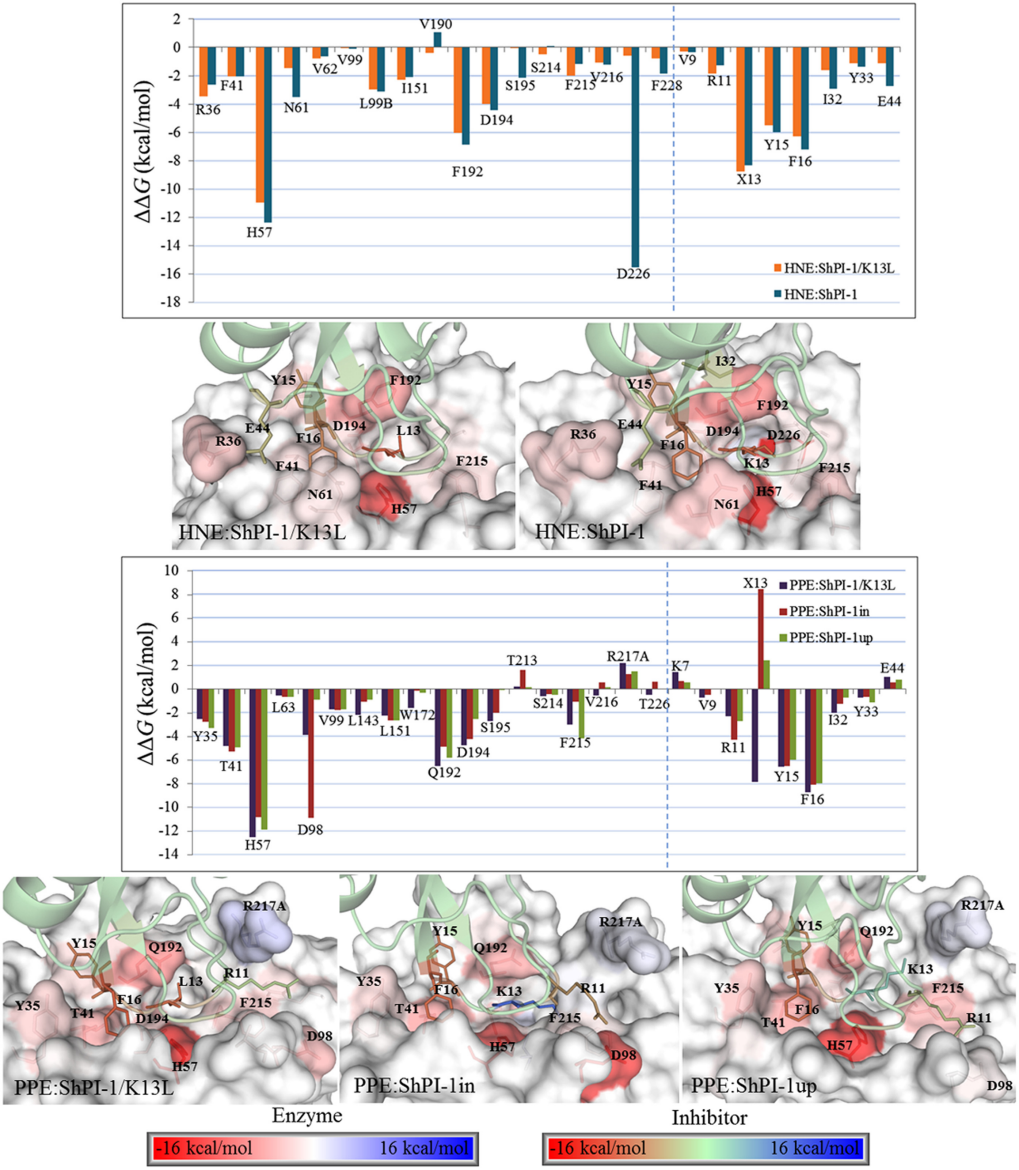
Per-Residue Energy Contributions to the Formation of the Studied Complexes. Residues identified as warm/hot-spots by *pr*EFED were selected for CAS, except for Cys, Pro, Ala and Gly. The vertical dashed lines separate the enzyme’s residues from those of the inhibitor. X13 stands for L13 or K13 depending on the complex. ΔΔ*G* = Δ*G*_*eff*_(native complex)-Δ*G*_*eff*_(mutated complex), therefore, a negative ΔΔ*G* value indicates a favorable energy contribution of the mutated residue to the complex formation. Conversely, a positive ΔΔ*G* value indicates and unfavorable contribution of the mutated residue. Error bars were not included since standard errors were always lesser than 5% of the mean values. A structural representation of the warm-/hot-spot residues at the interface of each complex is shown. Residues were colored according to their energy contribution to the complex formation, see color-gradient bars.

From the CAS results, three residues of the S1 subsite, i.e., H57, Q/F192 and D194, were identified as the most important common/equivalent hot-spots of PPE and HNE (ΔΔ*G≤*-4.0 kcal/mol) (Fig 11). Other S1 residues conserved in both elastases, i.e., S195, S214, F215 and V216, were also predicted as warm-/hot-spots (Fig 11), thereby reinforcing the importance of this subsite in the interaction with the inhibitors. Additionally, the residue D226, also located within the S1 subsite of HNE, had the largest energy contribution to the formation of the complex with the wild-type inhibitor (Fig 11). In fact, the mutated enzyme ENH/D226A would be unable to bind ShPI-1 due to an increase of ~16 kcal/mol in the Δ*G*_*eff*_ value (Fig 11). In contrast, the equivalent residue of PPE, T226, is not involved in the hypothetical interaction of this enzyme with ShPI-1. On the other hand, the residue at position 226 of both elastases is irrelevant for their interaction with ShPI-1/K13L (Fig 11). Hence, these results suggest that the mutated enzymes PPE/T226A and HNE/D226A display a PPE-like behavior against both inhibitor variants. For ShPI-1 and ShPI-1/K13L, highly-favorable energy contributions were predicted for the P1 site residues in all complexes except for both conformations of the PPE:ShPI-1 complex (Fig 11), which is not actually formed [7]. This result is consistent with the crucial importance of the P1 site residue in the interaction of canonical inhibitors with target proteases [13]. Interestingly, the energy contribution of K13 is less unfavorable in the ‘up’ conformation than in the ‘in’ conformation, in accordance with its lower polar-desolvation penalty when adopting the former conformation. Finally, warm-/hot-spots outside the S1:P1 interfaces of the complexes were also identified (see S2 Text). Remarkably, residue E44(P31’) lying outside the binding loops of the inhibitors was identified as a hot-spot at the interfaces of the HNE complexes (Fig 11).

To clarify the origin of the energetic contribution of these warm/hot-spots residues in terms of their pair-wise interactions with neighboring residues, the *pw*EFED protocol was employed. Residues D226 of HNE and K13 of ShPI-1 were predicted as the pair of interacting residues with the largest energy contribution to the HNE:ShPI-1 complex formation (Δ*G*_*D226*, *K13*_ = -26.87 kcal/mol). This result confirms the importance of the hydrogen bonds and salt bridge formed between the previous residues (Fig 4B) for the binding of the wild-type inhibitor to HNE. In agreement with the results obtained through the *pr*EFED and CAS approaches, the negligible energy contribution of D226 of HNE to the binding of ShPI-1/K13L arises from its weak pair-wise interaction with L13 at the P1 site (Δ*G*_*D226*, *K13*_ = -0.37 kcal/mol). A similar result explains the slight contribution of T226 of PPE in the interaction of this enzyme with both inhibitor variants (Δ*G*_*T226*, *K13in*_ = -0.07 kcal/mol, Δ*G*_*T226*, *K13up*_ = 0, Δ*G*_*T226*, *L13*_ = -0.61 kcal/mol). Interestingly, we also observed a favorable pair-wise interaction between E44 (P31’) of either ShPI-1 or ShPI-1/K13L and R36 of HNE (Δ*G*_*R36*, *E44*_ = -3.65 kcal/mol and -5.30 kcal/mol, respectively) due to the hydrogen bond and salt bridge formed between these residues (S3 and S4 Tables).

Interestingly, we also predicted that the total energy contribution of L13 due to pair-wise interactions with the PPE residues (-33.02 kcal/mol) is less than that of K13 in the PPE:ShPI-1*in* complex (-36.00 kcal/mol). This counterintuitive result highlights the impossibility of correlating the strength of pair-wise interactions with the complex affinity without taking into account the polar-desolvation penalty effects. Conversely, the pair-wise energy contribution of K13 in the PPE:ShPI-1*up* complex (-25.72 kcal/mol) is smaller than those of L13 and K13 in the PPE:ShPI-1/K13L and PPE:ShPI-1*in* complexes, respectively, in accordance with the reduced number of contacts established by K13 in the ‘up’ conformation (Fig 6B). It is noteworthy that the K13L mutation at the P1 site caused changes in the pair-wise interaction energies between residues outside the S1:P1 interface (data not shown). The occurrence of such changes spread over the entire complex interfaces, but mostly at the P2-P7 sites of the inhibitor and the corresponding subsites of the enzyme, helps understand why the PPE:ShPI-1/K13L complex possesses a larger pair-wise energy contribution compared to the PPE:ShPI-1*in* complex, even though L13 establishes weaker pair-wise interactions than K13. For the HNE complexes, pair-wise interaction energies of -33.00 kcal/mol and -68.98 kcal/mol were predicted for L13 and K13, respectively. However, the energy contribution of the latter residue is greatly diminished by its large polar-desolvation penalty (Fig 9). In addition, changes in the pair-wise interaction energies outside the S1:P1 interface, especially comprising the Sn’ and Pn’ sides of HNE and both inhibitor variants, respectively, (data not shown) caused the more favorable van der Waals energy of the HNE:ShPI-1/K13L complex with respect to the HNE:ShPI-1 complex (see Table 2).

## Discussion

### Position 226 determines the differential specificity of HNE and PPE for ShPI-1

The structural and energetic analyses performed in this work pointed out the critical role of position 226 of HNE and PPE in determining their ability to interact or not with the wild-type inhibitor ShPI-1. According to 3D structure model of the HNE:ShPI-1 complex, the residue D226 at the S1 subsite of HNE forms relatively stable hydrogen bonds and a salt bridge with the basic residue K13 at the P1 site of ShPI-1 (Fig 4B). This interaction is essential to overcome the high polar-desolvation penalty of the K13 residue (Fig 9). Furthermore, these predictions suggested that the enzyme variant ENH/D226A would be unable to bind ShPI-1 (Fig 11), thereby proposing an experimental approach to demonstrate the crucial role of the D226-K13 interaction in the complex formation.

The presence of D226 at the bottom of the S1 subsite of HNE would also explain the interaction of this enzyme with other canonical inhibitors bearing Lys or Arg at the P1 site, such as BPTI and the Kazal-type inhibitor CmpI-II, respectively [20, 74]. Indeed, the occurrence of close contacts (≤4 Å) between D226 and R12(P1) of the latter has been proposed before based on the analysis of a 3D-structure model of the HNE:CmPI-II complex [74]. Furthermore, in accordance with our predictions based on a GB model, a previous study also assessed the high desolvation penalties of ligands with positively-charged moieties at the P1 site upon their binding to thrombin, using quantum chemical calculations of solvation energies. A similar role to that of D226 was also suggested for residue D189 of thrombin in the compensation of the desolvation penalties of positively-charged moieties at the P1 site [75].

Unlike HNE, PPE has a Thr residue at position 226 that is unable to interact with K13 of the wild-type ShPI-1 (Fig 6). The previous fact may preclude the formation of the PPE:ShPI-1*in* complex, since the high polar-desolvation penalty associated with the insertion of K13 into the S1 subsite of PPE is not compensated by strong pair-wise interactions. An alternative ‘up’ conformation of K13 was sampled during the 125 ns MD simulation of the PPE:ShPI-1 complex (Fig 5). Interestingly, a similar conformation has been observed in the crystal structures of ShPI-1 and BPTI in complexes with bovine chymotrypsin (PDB: 3T62 and 1CBW, respectively), another SP with a hydrophobic S1 subsite [7, 15]. Of note, S217 at the entrance of the S1 subsite of chymotrypsin helps stabilize the ‘up’ conformation of a Lys residue at the P1 site through the formation of a hydrogen bond [7, 15]. In contrast, our predictions revealed the lack of strong interactions of K13 of ShPI-1 with the residues at the entrance of the S1 subsite of PPE. In particular, the absence of a hydrogen bond between K13(**NZ**) and S217(O) in the PPE:ShPI-1*up* complex (S6 Table), was corroborated, which is consistent with previous suggestions [7].

On the other hand, the results obtained from the MD simulations of the disrupted complexes also revealed the importance of the long-ranged electrostatic interaction between D226 of HNE and K13 of ShPI-1 in the binding process. In fact, upon an initial interface disruption in the HNE:ShPI-1 complex, the distance between both residues decreased from ~5.7 Å to an equilibrium distance of ~2.7 Å during the simulation time (Fig 7), which suggests that the D226-K13 attraction may be an essential driven force for the insertion of K13 side-chain into the enzyme S1 subsite. Conversely, the absence of long-range interactions between K13 and T226 of PPE is likely to preclude the formation of the PPE:ShPI-1*in* complex (Fig 7).

Our results strongly suggest the importance of the residue at position 226 of HNE and PPE in determining whether a positively-charged group of the inhibitor can be accommodated into the S1 subsite of these enzymes. In addition, the structural characteristics of the S1 subsite of PPE hamper the stabilization of inhibitor’s Lys residues at the entrance of this subsite. We suggest that these facts are the underlying causes of the experimentally demonstrated large difference in the affinity of ShPI-1 for HNE and PPE.

### The lower polar-desolvation penalty contributes to the higher affinity of ShPI-1/K13L for both elastasesd

The higher affinity of ShPI-1/K13L for PPE and HNE compared to that of ShPI-1 depends to a great extent on the lower polar-desolvation penalties associated with the binding of the former inhibitor to both enzymes (Table 2). In fact, this is the only energy component explaining the higher affinity of ShPI-1/K13L for HNE (Table 2). On the other hand, the PPE:ShPI-1/K13L complex has both stronger pair-wise interactions and a less unfavorable polar-desolvation penalty than the PPE:ShPI-1*in* complex. Interestingly, the stronger pair-wise van der Waals interactions of ShPI-1/K13L with PPE compared to ShPI-1 are not directly caused by its more hydrophobic P1 site residue, but rather by other neighboring residues at the Pn side of its primary binding loop, e.g., V9(P5), G10(P4) and, especially, R11(P3).

The well-known preference of elastases for aliphatic residues at the P1 site [23, 76] has been usually attributed to the relative hydrophobicity of their S1 subsites [22] (S1 Fig). Our results showed that this hydrophobicity should be understood as the lack of residues at key positions capable of establishing pair-wise interactions with polar P1 site residues, strong enough to overcome the highly unfavorable polar-desolvation penalties of the latter. Accordingly, the hydrophobicity of a binding site cannot be viewed as an average property mainly determined by the nature of its most abundant residues. In fact, the sole difference at position 226 has a great impact on the binding of HNE and PPE to PIs bearing basic residues at the P1 site; although, the S1 subsites of both enzymes have been commonly described as hydrophobic [22].

### P3, P2’ and P3’ sites as important positions for modulating the inhibitor specificity and affinity for elastases

As expected for a canonical serine protease inhibitor, the warm-/hot-spots residues of ShPI-1 and ShPI-1/K13L in their interaction with both elastases were identified mainly within the primary and secondary binding loops [13]. Interestingly, from all the previously identified residues, those at the P3, P1, P2’ and P3’sites were predicted to interact with regions of HNE and PPE displaying high variability in their amino acid composition (S2 Table). Considering such differences, amino acid substitution at the previous sites could modify the specificity of the inhibitor toward HNE or PPE. This has been experimentally confirmed at least for the P1 site of ShPI-1 and suggested for the P3 site of this inhibitor based on a previous structural analysis [7]. Moreover, our predictions show that R11 at P3 of ShPI-1 has more favorable energy contribution in the PPE complexes, mainly due to the presence of the negatively-charged residue D98 within the S3 subsite of PPE (N99A in HNE) (see S1 Fig and Fig 11). Thus, mutations at this position may enhance the specificity of ShPI-1/K13L for HNE.

Besides the well-known involvement of the P1 site residue in the binding to all SPs, including elastases, residues Y15(P2’) and F16(P3’) were predicted to have large energy contributions, comparable to those of the P1 site in the three existing complexes analyzed here (Fig 11). This result underscores the critical role of the interactions at the S2’:P2’ and S3’:P3’ interfaces in elastase inhibition, although a subordinate role of the interactions at the Sn’ and Pn’ sides with respect to those at the Sn and Pn sides of SPs and their substrate-like inhibitors, respectively, has been previously referred [77]. It is also likely that both elastases display a preference for aromatic residues at P2’ and P3’ sites. Hence, unlike the S3 subsite, the different residue composition between the respective S2’ and S3’ subsites is not expected to promote differences in the specificities of HNE and PPE for inhibitor residues at P2’ and P3’ sites. The preference for aromatic residues at P2’ and P3’ sites could explain the higher affinity of ShPI-1 for HNE (*K*_*i*_ = 1.3·10^−9^ M) compared to BPTI (*K*_*i*_ = 3.5·10^−6^ M) [7, 20], since the latter possesses non-aromatic Arg and Ile residues at these subsites.

### E44: A hot-spot located outside the inhibitor’s standard binding loops

Apart from the residues of the primary and secondary binding loops, our predictions suggest a hot-spot residue outside both loops, i.e., E44(P31’), which is involved in a hydrogen bond and a salt bridge with R36 of HNE in the complexes with ShPI-1 and ShPI-1/K13L (S3 and S4 Tables). The polar interactions between these residues constitute the structural basis of their energy contribution to the complex formation (Fig 11).

To our knowledge, this is the first work reporting the putative involvement of a residue so distal from the binding loops of a BPTI-Kunitz domain in the interaction with a target protease. It is noteworthy that a previous report has suggested the impact of residues near E44 (K46 in BPTI), since mutations within the segment 39-RAKR-42, contiguous to the secondary binding loop of BPTI, influence the inhibitory activity of this inhibitor against HNE [78]. However, the influence of K46(P31’) on the interaction of BPTI with HNE has not been explored to date. On the other hand, according to our predictions, E44 has an unfavorable contribution to the formation of the complex with PPE (Fig 11). Hence, we hypothesize that amino acid substitution at this position would contribute to the design of ShPI-1/K13L variants with increased specificity for PPE.

## Conclusions

In this work the structural and energetic analyses of the interaction of ShPI-1 and ShPI-1/K13L with PPE and HNE were performed. Residue D226 was identified as structural hallmark of HNE for the interaction with inhibitors with basic chains at the P1 site. The lower polar-desolvation penalties associated with the binding of the ShPI-1/K13L to both elastases are also suggested to constitute the main energy component contributing to its larger affinity for these enzymes compared to that of the wild-type inhibitor ShPI-1. In addition, we carried out an extensive study of the interactions and the energy contributions of the residues at the complex interfaces. This analysis confirmed the importance of residues of the primary and secondary binding loops of BPTI-Kunitz inhibitors in their interaction with SPs and, especially, that of the P1 site residue. In addition to this site, residues at the P3, P2’ and P3’ were predicted as important positions to modulate the specificity and/or affinity of the inhibitors for elastases. We also suggested for the first time that E44, a residue located at a distal position from the binding loops, has a significant energy contribution to the interaction with HNE. Overall, the methodology and results of this work will be valuable for the design of new ShPI-1 variants more potent and/or specific for either HNE or PPE.

## Supporting Information

S1 Fig Structural Alignment of PPE and HNE Shown at the Level of Their Respective Primary Sequences. The sequence identity between both proteins is ~39%. Asterisks have been placed under the conserved positions and black dots, under the residues belonging to the S1 subsite of each elastase. Chymotrypsinogen residue numbering has been adopted [22]. Residues depicted in red belong to the catalytic triad of SPs. Additionally; yellow rectangles have been used to highlight the conserved Cys residues. The structural alignment was carried out with Modeller v9.5.
(TIF)

S2 FigThermodynamic Cycle Leading to the Calculation of Polar-Desolvation Penaly of Solute Atom *i* upon Complex Formation ($\Delta G_{ds}^{i}$).RL, R and L stand for the complex, the receptor and the ligand, respectively. The blue box represents the solvent. Δ*E*_*el*_ is the electrostatic energy variation upon complex formation in vacuum. *G*_GB(X)_ represents the polar-solvation free energy of molecule X, where X stands for R, L or RL. Similarly, $G_{GB\left(X\right)}^{ii}$ represents the polar-solvation free energy of atom *i* belonging to molecule X. The partial charges of all atoms of R and L except for atom *i* (orange dot) were set to zero. The inner space of R and L is shown in white to suggest the absence of intra-solute electrostatic interactions. The iteration of the thermodynamic cycle for every particle *i* of the solute molecules leads to the calculation of Δ*G*_*ds*_ as indicated in the figure. Note, however, that two terms standing for cross-energies ($\Delta G_{GB}^{ij}$) between particles *i* and *j* within the same solute molecule (R or L) must be added to the self-energies ($\Delta G_{GB}^{ii}$).(TIF)

S3 Fig Side-Chain Rearrangement of Q192 of PPE in the Representative Structure of the PPE:ShPI-1/K13L Complex. The hydrogen bond Q192(**NE2**):C12(O) is shown as a yellow dashed line. The average donor-acceptor distance and the hydrogen bond occupancy are also depicted. Note that according to the predictions of the MD simulation performed with AMBER99SB, the side-chain of Q192 undergoes a rearrangement that enables the formation a hydrogen bond not present in the crystal structure of the PPE:ShPI-1/K13L (PDB: 3UOU). Note also that the side-chain conformation of Q192 in the crystal structure prevents the formation of a hydrogen bond with G14(P1’).
(TIF)

S4 Fig Hydrogen Bonds Formed by Residues R18 and G35 of ShPI-1/K13L and the Residue at Position 61 of PPE and HNE in the Representative Structures of Both Complexes. Hydrogen bonds are represented by yellow dashed lines. The average donor-acceptor distance is also depicted.
(TIF)

S5 Fig Interactions between Residue E44 of ShPI-1/K13L and R36 of HNE and R61 of PPE in the Representative Structures of Both Complexes. The predicted hydrogen bonds and/or salt bridges are represented by blue dashed lines. The average donor-acceptor distance is also depicted.
(TIF)

S1 Table Comparison of the van der Waals Contacts and Hydrogen Bonds at the Interfaces of the PPE:ShPI-1/K13L Crystal and Representative Structures. Van der Waals contacts were determined with a cutoff radius of 4 Å. For hydrogen bonds, the geometric constraints were *i*) a distance ≤3.5 Å between the donor and the acceptor and *ii*) an acceptor-donor-hydrogen angle ≤30°. The names of the donor and acceptor atoms, as well as the donor-acceptor distance and the hydrogen bond occupancy during the productive MD simulation are shown in red. The names of PPE residues involved in Van der Waals contacts conserved in both complexes are shown in bold style.
(DOCX)

S2 Table Van der Waals Contacts at the Interfaces of the Thee Existing Complexes. Van der Waals contacts were determined with a cutoff radius of 4 Å.
(DOCX)

S3 Table Hydrogen Bonds at the Interfaces of the Three Existing Complexes. Hydrogen bonds with an occupancy ≥30% at least in one of the four interfaces are shown.
(DOCX)

S4 Table Salt Bridges at the Complex Interfaces of the Three Existing Complexes. Salt bridges were defined as the interaction between oppositely-charged residues of different protein chains within a distance ≤ 4 Å, at least in one snapshot of each productive MD simulation.
(DOCX)

S5 Table Van der Waals Contacts at the Interfaces of the ‘In’ and ‘Up’ Conformations of the PPE:ShPI-1 Complex. Van der Waals contacts were determined with a cutoff radius of 4 Å.
(DOCX)

S6 Table Hydrogen Bonds and Salt Bridges at the Interfaces of the ‘In’ and ‘Up’ Conformations of the PPE:ShPI-1 Complex. Hydrogen bonds with an occupancy ≥30% at least in one of the two interfaces are shown. Other interactions not fulfilling the previous condition are also shown for comparison with those of the three existing complexes.
(DOCX)

S7 Table Standard MM-GBSA Energy Components for the Studied Complexes. (DOCX)

S8 Table Main Per-Residue Energy Contributions Calculated by *pr*EFED for the HNE:ShPI-1 and HNE:ShPI-1/K13L Complexes. Only those residues for which |Δ*G*_*res*_|≥1.0 kcal/mol and/or |Δ*G*_*sc*_|≥0.5 kcal/mol at least in one of the complexes are shown here. Note that E44 was included because of its interaction with R36, even though it does not fulfill the previous conditions.
(DOCX)

S9 Table Main Per-Residue Energy Contributions Calculated by *pr*EFED for the PPE:ShPI-1*in* and PPE:ShPI-1/K13L Complexes. Only those residues for which |Δ*G*_*res*_|≥1.0 kcal/mol and/or |Δ*G*_*sc*_|≥0.5 kcal/mol at least in one of the complexes are shown here. Note that T226 was included to compare its energy contribution wih that of D226 of HNE, even though it does not fulfill the previous conditions.
(DOCX)

S10 Table Main Per-Residue Energy Contributions Calculated by *pr*EFED for the PPE:ShPI-1*up* and PPE:ShPI-1/K13L Complexes. Only those residues for which |Δ*G*_*res*_|≥1.0 kcal/mol and/or |Δ*G*_*sc*_|≥0.5 kcal/mol at least in one of the complexes are shown here. Note that T226 was included to compare its energy contribution wih that of D226 of HNE, even though it does not fulfill the previous conditions.
(DOCX)

S1 Text Analysis of the Polar Interactions Outside the S1:P1 Interfaces in the Three Existing Complexes. (DOCX)

S2 Text Per-Residue Energy Contributions outside the S1:P1 Interfaces of the Studies Complexes. (DOCX)
