# Supplementary figures and images for: Polar Desolvation and Position 226 of Pancreatic and Neutrophil Elastases Are Crucial to their Affinity for the Kunitz-Type Inhibitors ShPI-1 and ShPI-1/K13L
Source: PLoS One. 2015 Sep 15;10(9):e0137787. doi: 10.1371/journal.pone.0137787 (PMC4570792; doi:10.1371/journal.pone.0137787)

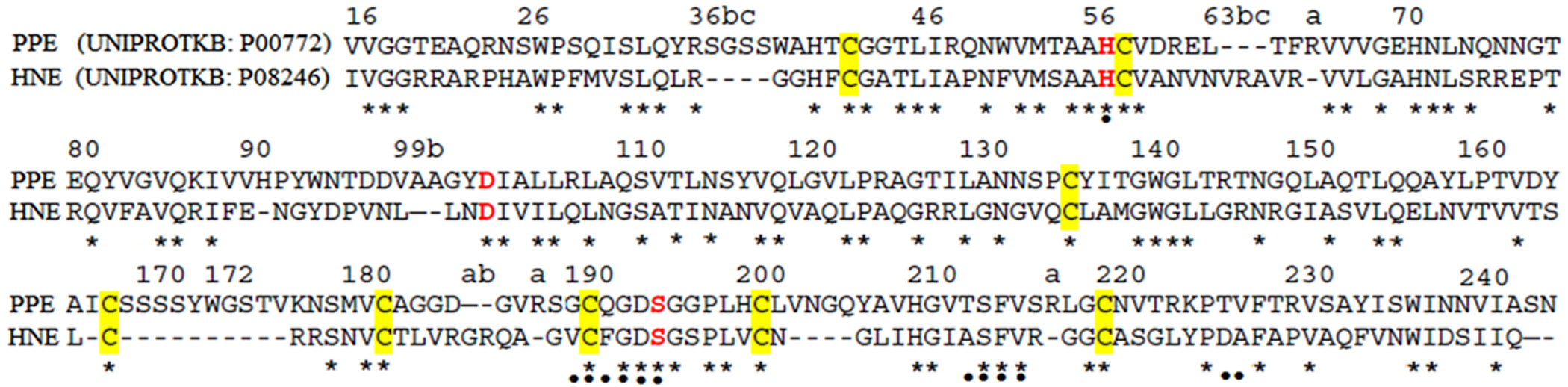

Supplement: S1 Fig — The sequence identity between both proteins is ~39%. Asterisks have been placed under the conserved positions and black dots, under the residues belonging to the S1 subsite of each elastase. Chymotrypsinogen residue numbering has been adopted [22]. Residues depicted in red belong to the catalytic triad of SPs. Additionally; yellow rectangles have been used to highlight the conserved Cys residues. The structural alignment was carried out with Modeller v9.5. (TIF) [file pone.0137787.s001.tif]

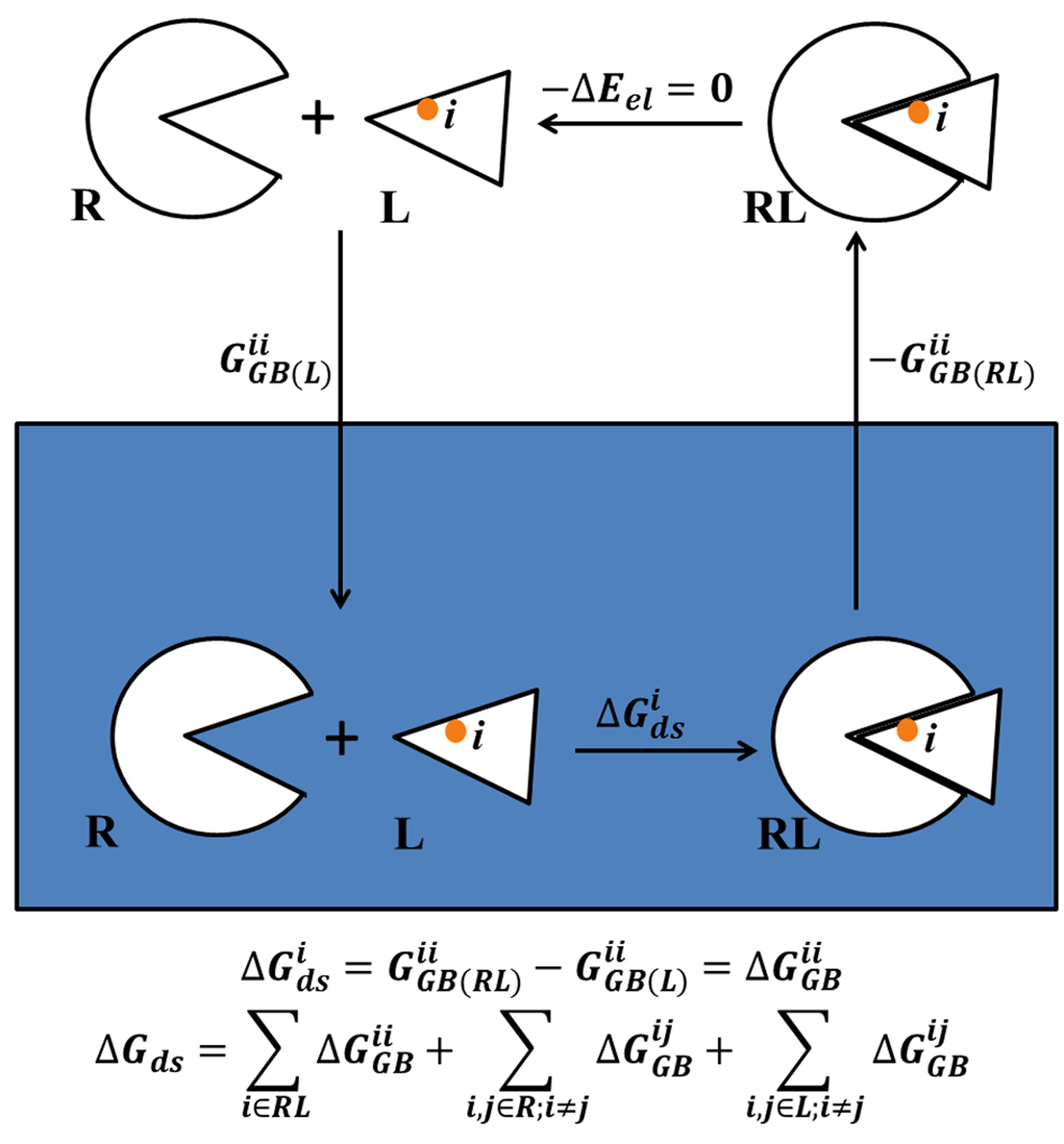

Supplement: S2 Fig — RL, R and L stand for the complex, the receptor and the ligand, respectively. The blue box represents the solvent. ΔE el is the electrostatic energy variation upon complex formation in vacuum. G GB(X) represents the polar-solvation free energy of molecule X, where X stands for R, L or RL. Similarly, GGB(X)ii represents the polar-solvation free energy of atom i belonging to molecule X. The partial charges of all atoms of R and L except for atom i (orange dot) were set to zero. The inner space of R and L is shown in white to suggest the absence of intra-solute electrostatic interactions. The iteration of the thermodynamic cycle for every particle i of the solute molecules leads to the calculation of ΔG ds as indicated in the figure. Note, however, that two terms standing for cross-energies (ΔGGBij) between particles i and j within the same solute molecule (R or L) must be added to the self-energies (ΔGGBii). (TIF) [file pone.0137787.s002.tif]

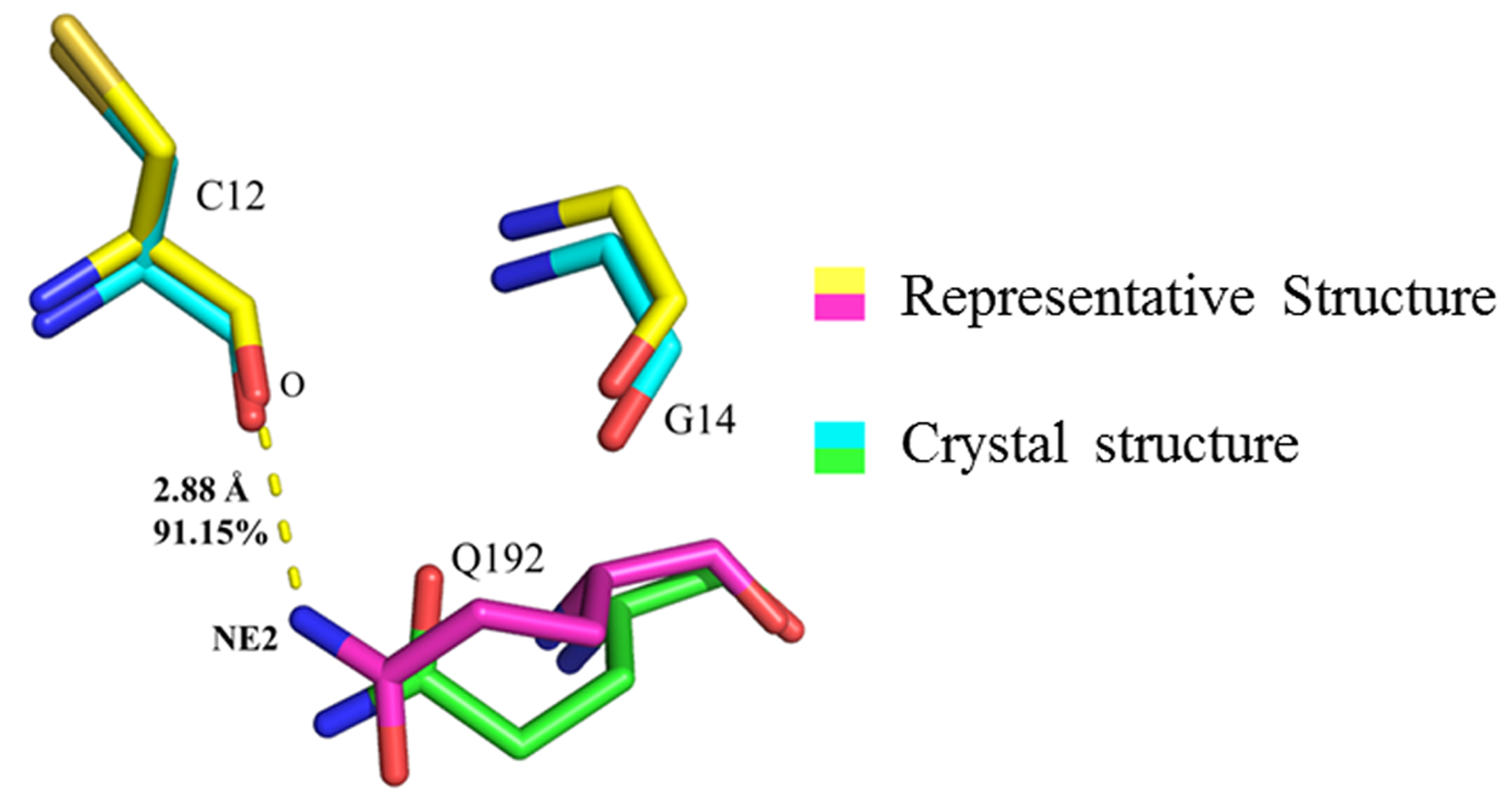

Supplement: S3 Fig — The hydrogen bond Q192(NE2):C12(O) is shown as a yellow dashed line. The average donor-acceptor distance and the hydrogen bond occupancy are also depicted. Note that according to the predictions of the MD simulation performed with AMBER99SB, the side-chain of Q192 undergoes a rearrangement that enables the formation a hydrogen bond not present in the crystal structure of the PPE:ShPI-1/K13L (PDB: 3UOU). Note also that the side-chain conformation of Q192 in the crystal structure prevents the formation of a hydrogen bond with G14(P1’). (TIF) [file pone.0137787.s003.tif]

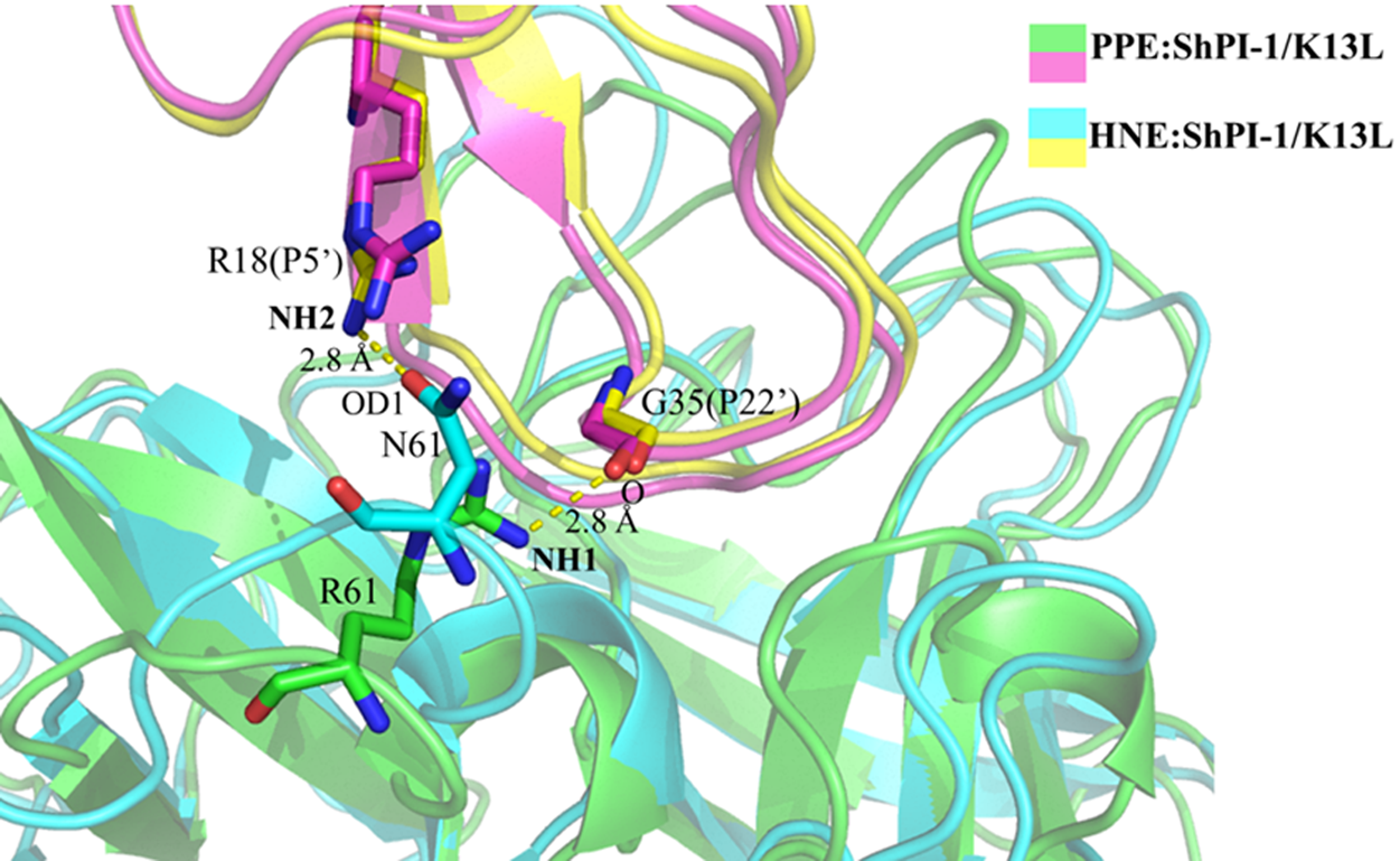

Supplement: S4 Fig — Hydrogen bonds are represented by yellow dashed lines. The average donor-acceptor distance is also depicted. (TIF) [file pone.0137787.s004.tif]

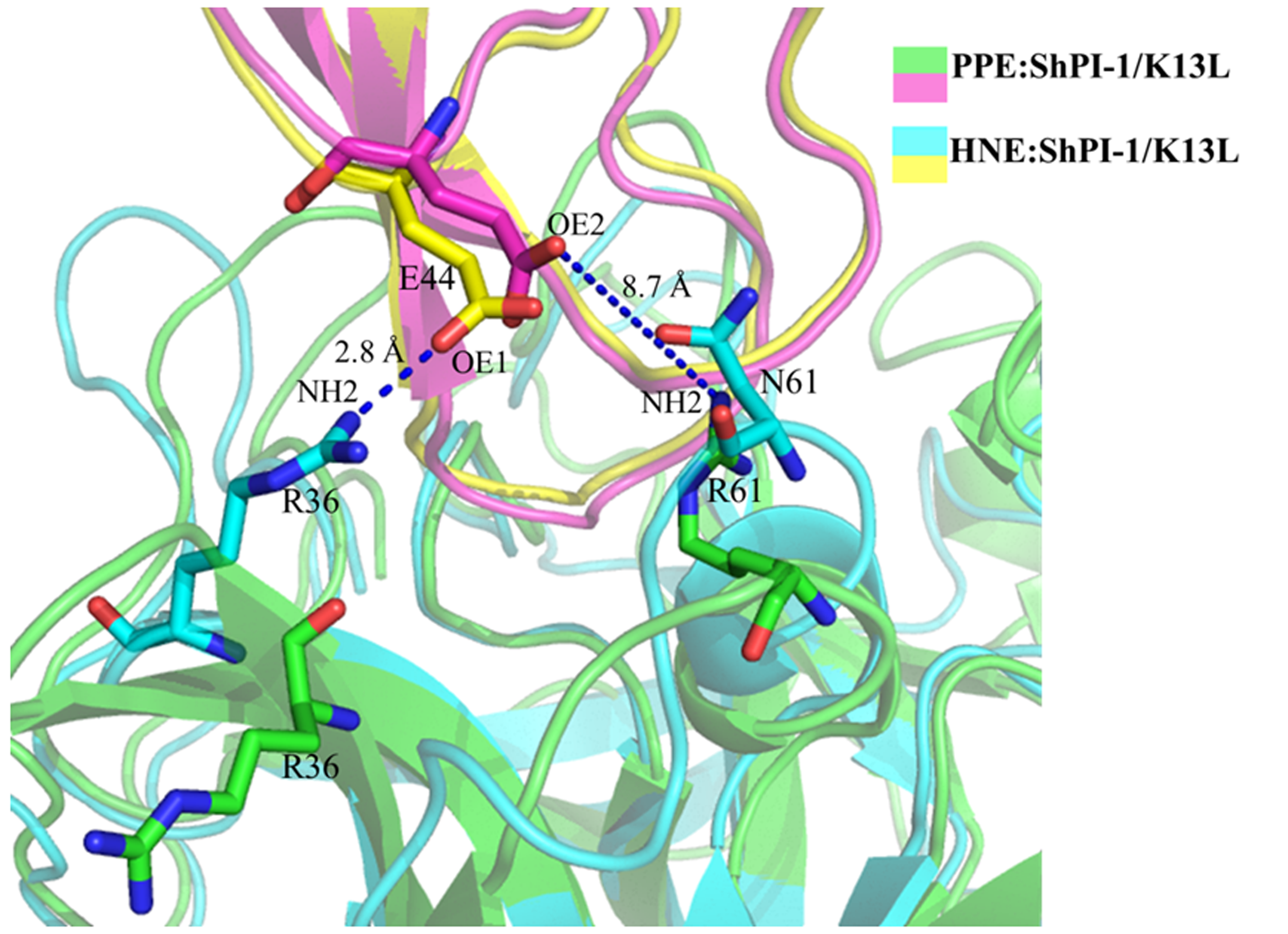

Supplement: S5 Fig — The predicted hydrogen bonds and/or salt bridges are represented by blue dashed lines. The average donor-acceptor distance is also depicted. (TIF) [file pone.0137787.s005.tif]
