# Supplementary material for: Polar Desolvation and Position 226 of Pancreatic and Neutrophil Elastases Are Crucial to their Affinity for the Kunitz-Type Inhibitors ShPI-1 and ShPI-1/K13L
Source: PLoS One. 2015 Sep 15;10(9):e0137787. doi: 10.1371/journal.pone.0137787 (PMC4570792; doi:10.1371/journal.pone.0137787)
Supplement: S1 Table — Van der Waals contacts were determined with a cutoff radius of 4 Å. For hydrogen bonds, the geometric constraints were i) a distance ≤3.5 Å between the donor and the acceptor and ii) an acceptor-donor-hydrogen angle ≤30°. The names of the donor and acceptor atoms, as well as the donor-acceptor distance and the hydrogen bond occupancy during the productive MD simulation are shown in red. The names of PPE residues involved in Van der Waals contacts conserved in both complexes are shown in bold style. (DOCX) [file pone.0137787.s006.docx]

| **ShPI-1/K13L** | | **PPE (PDB: 3UOU)** | **PPE (representative structure)** |
| --- | --- | --- | --- |
| **P6** | K8 | R217A |  |
| **P5** | V9 | **Q192** | **Q192** |
| **P4** | G10 | R217A |  |
| **P3** | R11 | D98 NH1-OD1 [5.25]^a^, **A99A**, **T175**,**F215**, **V216** O-N [2.82], **R217A** | D98 NH1-OD2 [3.07, 18.34%]^a^, V99, **A99A**,W172, **T175**, **F215**, **V216** O-N [2.98, 99.35%], S217, **R217A** |
| **P2** | C12 | **H57**,**V99**, **Q192** O-NE2 [6.09]^a^, **S214** | **H57**, **V99**, **Q192** O-NE2 [2.88, 91.15%]^a^, **S214**, F215 |
| **P1** | L13 | **H57, G190,C191, Q192, G193** O-N [2.73] **, D194, S195** O-N [3.04]**, T213, S214** N-O [3.33]**, V216** | **H57, G190, C191, Q192 , G193** O-N [2.75, 94.00%]**, D194**, **S195** O-N [3.05, 93.45%]**, T213, S214** O-N [3.33, 28.64%]**,** F215, **V216**, T226 |
| **P1’** | G14 | **T41, H57, Q192, G193, S195** | **T41,** C42 **,H57, Q192 ,G193, S195** |
| **P2’** | Y15 | **H40, T41** N-O [3.00]; O-OG1[2.88];  N-OG1 [3.50], **L143, L151, G193** | Y35, **H40,** **T41** N-O [2.89, 35.98%]; O-OG1[2.71, 76.71%]; N-OG1 [3.44, 51.12%], **L143**, Q150, **L151** ,Q192, **G193** |
| **P3’** | F16 | **T41, C58, R61** | Y35, **T41**, H57**, C58**, **R61**, L63 |
| **P4’** | P17 | **Y35** | **Y35** |
| **P5’** | R18 | **R61** | **R61** |
| **P19’** | I32 | **Q192** | L143, **Q192** |
| **P21’** | G34 | **H57, Q192** | **H57, Q192** |
| **P22’** | G35 | **H57**, **R61** O-NH1 [2.77] | **H57**, T96, **R61** O-NH1 [2.84, 78.66%] |
| **P23’** | C38 | **T96, V99** | **H57**, W94 ,**T96**, **V99** |
| **P24’** | G37 | **T96** N-O [2.91] | **T96** N-O [3.85, 24.09%], D97 |

^a^Hydrogen bonds not observed in both complexes. Note that the names of the donor and acceptor atoms and their distance are depicted in black when the hydrogen bond was not actually formed.
