# Supplementary material for: Polar Desolvation and Position 226 of Pancreatic and Neutrophil Elastases Are Crucial to their Affinity for the Kunitz-Type Inhibitors ShPI-1 and ShPI-1/K13L
Source: PLoS One. 2015 Sep 15;10(9):e0137787. doi: 10.1371/journal.pone.0137787 (PMC4570792; doi:10.1371/journal.pone.0137787)
Supplement: S2 Table — Van der Waals contacts were determined with a cutoff radius of 4 Å. (DOCX) [file pone.0137787.s007.docx]

|  | **PPE:ShPI-1/K13L** | | **HNE:ShPI-1** | | **HNE:ShPI-1/K13L** | |
| --- | --- | --- | --- | --- | --- | --- |
| **Site** | **I** | **E** | **I** | **E** | **I** | **E** |
| **5** | V9 | Q192 | V9 | F192 | V9 | F192 |
| **4** | G10 | R217A | G10 | F192 | - |  |
| **3** | R11 | D98, V99, A99A, W172, T175, F215, V216, S217 R217A | R11 | V99, L99B, F215, V216 | R11 | V99, L99B, F215,  V216, R217 |
| **2** | C12 | H57, V99, Q192, S214, F215 | C12 | H57, L99B  F192, S214  F215, V216 | C12 | H57, L99B, F192, S214, F215, V216 |
| **1** | L13 | H57, G190, C191, Q192, G193, D194, S195, T213, S214, F215, V216, T226 | K13 | H57, V190, C191, F192, G193, D194, S195, A213  S214, F215, V216, D226, A227 | L13 | H57, V190, C191, F192, G193, D194  S195, A213, S214, F215, V216 |
| **1’** | G14 | T41, C42, H57, Q192, G193, S195 | G14 | F41, C42, H57, C58, F192, G193, S195 | G14 | F41, C42, H57, F192, G193, S195 |
| **2’** | Y15 | Y35, H40, T41, L143, Q150, L151  Q192, G193 | Y15 | H40, F41, L143, I151, F192, G193 | Y15 | H40, F41, L143, I151, F192, G193 |
| **3’** | F16 | Y35, T41, H57, C58, R61, L63 | F16 | F41, C58, N61, V62 | F16 | F41, H57  C58, V62 |
| **4’** | P17 | Y35 | - | - | - | - |
| **5’** | R18 | R61 | R18 | N61 | R18 | N61, V62 |
| **19’** | I32 | L143, Q192 | I32 | F192 | I32 | F192 |
| **20’** | Y33 | R61, Q192 | Y33 | N61 | - | - |
| **21’** | G34 | H57, Q192 | G34 | H57, F192 | G34 | H57, F192 |
| **22’** | G35 | H57, T96 | G35 | H57, C58  N61 | G35 | H57 |
| **23’** | C36 | H57, W94  T96, V99 | C36 | H57, Y94  P98, L99B | C36 | H57, Y94  P98, L99B |
| **24’** | G37 | T96, D97 | - | - | - | - |

^a^ E and I stand for the enzyme and the inhibitor, respectively.
