# Supplementary material for: Polar Desolvation and Position 226 of Pancreatic and Neutrophil Elastases Are Crucial to their Affinity for the Kunitz-Type Inhibitors ShPI-1 and ShPI-1/K13L
Source: PLoS One. 2015 Sep 15;10(9):e0137787. doi: 10.1371/journal.pone.0137787 (PMC4570792; doi:10.1371/journal.pone.0137787)
Supplement: S3 Table — Hydrogen bonds with an occupancy ≥30% at least in one of the four interfaces are shown. (DOCX) [file pone.0137787.s008.docx]

|  | **HNE** | | | | **PPE** | | |
| --- | --- | --- | --- | --- | --- | --- | --- |
| **Site** | **I^a^** | **E^a^** | **ShPI-1**  **Occ (%)^b^** | **K13L^c^**  **Occ (%)** | **I** | **E** | **K13L**  **Occ (%)** |
| P3 | R11(O)^d^ | V216(**N**)^d^ | 98.55 | 98.85 | R11(O) | V216(**N**) | 99.35 |
|  | R11(**NH1**) | V99(O) | 40.33 | 29.14 | R11(**NH1**) | D98(OD1) | 9.35 |
|  | - | - | - | - | R11(**NH1**) | D98(OD2) | 18.34 |
| P2 | - | - | - | - | C12(O) | Q192(**NE2**) | 91.15 |
| P1 | X13(O)^e^ | G193(**N**) | 72.86 | 93.30 | X13(O) | G193(**N**) | 94.00 |
|  | X13(O) | S195(**N**) | 69.97 | 82.76 | X13(O) | S195(**N**) | 93.45 |
|  | X13(**N**) | H57(NE2) | 44.93 | 20.39 | X13(**N**) | H57(NE2) | 28.14 |
|  | X13(**N**) | S214(O) | 8.00 | 40.53 | X13(**N**) | S214 (O) | 28.64 |
|  | K13(**NZ**) | S214(O) | 42.73 | - | - | - | - |
|  | K13(**NZ**) | D226(OD1) | 54.82 | - | - | - | - |
|  | K13(**NZ**) | D226(OD2) | 45.98 | - | - | - | - |
| P2’ | Y15(**N**) | F41(O) | 31.18 | 73.21 | Y15(**N**) | T41(O) | 35.98 |
|  | - | - | - | - | Y15(**N**) | T41(OG1) | 51.12 |
|  | - | - | - | - | Y15(O) | T41(**OG1**) | 76.71 |
| P5’ | R18(**NH2**) | N61(OD1) | 48.83 | 43.48 | - | - | - |
| P22’ | G35(O) | N61(**ND2**) | 38.03 | 0.10 | G35(O) | R61(**NH1**) | 78.66 |
| P31’ | E44(OE1) | R36(**NH2**) | 16.94 | 30.93 | - | - | - |

^a^I and E stand for the residues of the inhibitor and the enzyme, respectively.

^b^The hydrogen bond stability is expressed as occupancy (occ.), i.e., the percentage of snapshots fulfilling the geometric criteria for the interaction occurrence with respect to all the snapshots extracted from each productive MD simulation.

^c^Abbreviation of ShPI-1/K13L

^d^Donor and acceptor atom names are indicated between parentheses in bold and plain styles, respectively.

^e^X13 stands for either K13 or L13, depending on the complex.
