# Supplementary material for: Polar Desolvation and Position 226 of Pancreatic and Neutrophil Elastases Are Crucial to their Affinity for the Kunitz-Type Inhibitors ShPI-1 and ShPI-1/K13L
Source: PLoS One. 2015 Sep 15;10(9):e0137787. doi: 10.1371/journal.pone.0137787 (PMC4570792; doi:10.1371/journal.pone.0137787)
Supplement: S4 Table — Salt bridges were defined as the interaction between oppositely-charged residues of different protein chains within a distance ≤ 4 Å, at least in one snapshot of each productive MD simulation. (DOCX) [file pone.0137787.s009.docx]

|  | **HNE** | | | | **PPE** | | |
| --- | --- | --- | --- | --- | --- | --- | --- |
| **Site** | **I^a^** | **E^a^** | **ShPI-1**  **Dist. (Å)^b^** | **K13L^c^ Dist. (Å)** | **I** | **E** | **K13L**  **Dist. (Å)** |
| P3 | **-** | **-** | **-** | **-** | R11  (**NH1,NH2**) | D98  (OD1,OD2) | 5.72±1.32 |
| P1 | K13  (**NZ**)^d^ | D226  (OD1,OD2)^d^ | 2.73±0.13^e^ | - | - | - | - |
| P31’ | E44  (OE1,OE2) | R36  (**NH1,NH2**) | 6.02±2.53 | 5.72±2.71 | E44  (OE1,OE2) | R61  (**NH1**,**NH2**) | 9.60±1.10 |

^a^I and E stand for the residues of the inhibitor and the enzyme, respectively.

^b,c^Abbreviations of distance and ShPI-1/K13L, respectively.

^d^The names of the positively- and negatively-charged atoms involved in the salt bridge interaction are indicated between parentheses in bold and plain styles, respectively.

^e^Mean value ±standard deviation is shown.
