# Supplementary material for: Polar Desolvation and Position 226 of Pancreatic and Neutrophil Elastases Are Crucial to their Affinity for the Kunitz-Type Inhibitors ShPI-1 and ShPI-1/K13L
Source: PLoS One. 2015 Sep 15;10(9):e0137787. doi: 10.1371/journal.pone.0137787 (PMC4570792; doi:10.1371/journal.pone.0137787)
Supplement: S5 Table — Van der Waals contacts were determined with a cutoff radius of 4 Å. (DOCX) [file pone.0137787.s010.docx]

|  | **PPE:ShPI-1in^a^** | | **PPE:ShPI-1up^a^** | |
| --- | --- | --- | --- | --- |
| **Site** | **I^b^** | **E^b^** | **I** | **E** |
| **5** | V9 | Q192 | V9 | - |
| **4** | G10 | V216 | G10 | - |
| **3** | R11 | D97, D98,V99, A99A, F215, V216 | R11 | A99A, T175, M180, F215 |
| **2** | C12 | H57, V99, Q192, S195, S214, F215, V216 | C12 | H57, V99 |
| **1** | K13 | H57, G190, C191, Q192, G193, D194 S195, T213, S214, F215 V216,T226 | K13 | H57, Q192, G193, S214, F215, V216, R217A |
| **1’** | G14 | T41, C42, H57, Q192, G193, S195 | G14 | T41,C42, H57, Q192, S195 |
| **2’** | Y15 | Y35, H40, T41, L143,  Q150, L151, Q192, G193 | Y15 | T41, L143, Q150, L151, Q192, G193 |
| **3’** | F16 | Y35, T41, H57, C58, L63, F65 | F16 | Y35, T41, C58, L63 |
| **4’** | P17 | Y35 | P17 | Y35 |
| **5’** | - | - | R18 | R61 |
| **19’** | I32 | Q192 | I32 | Q192 |
| **21’** | G34 | H57, Q192 | G34 | H57, Q192 |
| **22’** | G35 | H57 | G35 | H57 |
| **23’** | C36 | H57, T96, V99 | C36 | H57, V99 |
| **24’** | G37 | T96 | G37 | T96 |

^a^PPE:ShPI-1in stands for the conformation of the PPE:ShPI-1 complex with the P1 site side-chain inserted into the S1 subsite of PPE, whereas PE:ShPI-1up represents a conformation with the P1 site side-chain bent at the entrance of the S1 subsite.

^b^E and I stand for the enzyme and the inhibitor, respectively.
