# Supplementary material for: Polar Desolvation and Position 226 of Pancreatic and Neutrophil Elastases Are Crucial to their Affinity for the Kunitz-Type Inhibitors ShPI-1 and ShPI-1/K13L
Source: PLoS One. 2015 Sep 15;10(9):e0137787. doi: 10.1371/journal.pone.0137787 (PMC4570792; doi:10.1371/journal.pone.0137787)
Supplement: S6 Table — Hydrogen bonds with an occupancy ≥30% at least in one of the two interfaces are shown. Other interactions not fulfilling the previous condition are also shown for comparison with those of the three existing complexes. (DOCX) [file pone.0137787.s011.docx]

| **Hydrogen Bonds** | | | | |
| --- | --- | --- | --- | --- |
|  | **PPE** | | | |
| **Site** | **I^a^** | **E^a^** | **ShPI-1in^b^**  **Occ. (%)^c^** | **ShPI-1up^b^**  **Occ. (%)** |
| **3** | R11(O)^d^ | V216**(N)^d^** | 96.45 | - |
|  | R11(**NH1**) | D98(OD1) | 42.13 | - |
|  | R11(**NH1**) | D98(OD2) | 31.83 | - |
|  | R11(**NH2**) | D98(OD1) | 30.28 | 0.55 |
| **2** | C12(O) | Q192(**NE2**) | 81.51 | - |
| **1** | K13(O) | S195(**N**) | 87.61 | 2.74 |
|  | K13(O) | G193(**N**) | 72.51 | 96.64 |
|  | K13(**N**) | H57(NE2) | 19.84 | 42.89 |
|  | K13(**N**) | S214 (O) | 25.14 | 0.62 |
|  | K13(**NZ**) | S214(O) | 5.80 | 0.62 |
|  | K13(**NZ**) | T226(OG1) | 1.20 | - |
| **1’** | G14(O) | Q192(**NE2)** | 3.40 | 66.79 |
| **2’** | Y15(O) | T41(**OG1**) | 46.28 | 16.67 |
|  | Y15(**N**) | T41(OG1) | 83.51 | 43.14 |
|  | Y15(**N**) | T41(O) | 5.25 | 52.28 |
| **22’** | G35(O) | R61(**NH1**) | 29.69 | - |
| **24’** | G37(O) | T96(**OG1)** | 7.55 | 36.55 |
| **Salt Bridges** | | | | |
| **Site** | **PPE** | | | |
|  | **I** | **E** | **ShPI-1in**  **Dist.^e^ (Å)** | **ShPI-1up**  **Dist. (Å)** |
| **3** | R11  (**NH1,NH2**)^d^ | D98  (OD1,OD2)^d^ | 4.66±0.93^e^ | 8.29±1.36 |
| **31’** | E44  (OE1, OE2) | R61  (**NH1**,**NH2**) | 8.64±2.08 | 10.96±1.88 |

^a^I and E stand for the residues of the inhibitor and the enzyme, respectively.

^b^ShPI-1in stands for the conformation of the PPE:ShPI-1 complex with the P1 site side-chain inserted into the S1 subsite of PPE, whereas ShPI-1up represents that with the P1 site side-chain bent at the entrance of the S1 subsite.

^c^The hydrogen bond stability is expressed as occupancy (occ.), i.e., the percentage of snapshots fulfilling the geometric criteria for the interaction occurrence with respect to all the snapshots extracted from each productive MD simulation.

^d^Donor and acceptor atom names are indicated between parentheses in bold and plain styles, respectively.

^e^Abbreviations of distance.

^d^The names of the positively- and negatively-charged atoms involved in the salt bridge interaction are indicated between parentheses in bold and plain styles, respectively.

^e^Mean value ±standard deviation is shown.
