# Supplementary material for: Polar Desolvation and Position 226 of Pancreatic and Neutrophil Elastases Are Crucial to their Affinity for the Kunitz-Type Inhibitors ShPI-1 and ShPI-1/K13L
Source: PLoS One. 2015 Sep 15;10(9):e0137787. doi: 10.1371/journal.pone.0137787 (PMC4570792; doi:10.1371/journal.pone.0137787)
Supplement: S7 Table — (DOCX) [file pone.0137787.s012.docx]

| Complex | Δ*E_gas_*  **(kcal/mol)** | Δ*G_SA_*  **(kcal/mol)** | Δ*G_GB/PB_*  **(kcal/mol)** | Δ*G_solv_*  **(kcal/mol)** | Δ*G_eff_*^d^  **(kcal/mol)** |
| --- | --- | --- | --- | --- | --- |
| PPE:ShPI-1in | -101.70±0.85 | -11.55±0.02 | 50.92±0.84 (2)^c^  58.94±0.82 (5)  53.55±0.85 (8)  40.61±0.88 (pb1)  56.79±0.80 (pb2)  58.81±0.81 (pb3) | 39.37±0.84 (2)  47.39±0.82 (5)  42.01±0.85 (8)  29.06±0.88 (pb1)  45.24±0.80 (pb2)  47.26±0.81 (pb3) | -62.33±0.14 (2)  -54.31±0.15 (5)  -59.69±0.13 (8)  -72.64±0.22 (pb1)  -56.46±0.17 (pb2)  -54.44±0.17 (pb3) |
| PPE:ShPI-1up | -63.28±0.70 | -11.10±0.01 | 20.34±0.66 (2)  24.00±0.65 (5)  18.91±0.67 (8)  22.73±0.69 (pb1)  21.43±0.67 (pb2)  22.05±0.67 (pb3) | 9.24±0.66 (2)  12.90±0.65 (5)  7.81±0.67 (8)  11.63±0.69 (pb1)  10.33±0.67 (pb2)  10.95±0.67 (pb3) | -54.04±0.10 (2)  -50.38±0.11 (5)  -55.47±0.10 (8)  -51.65±0.20 (pb1)  -52.95±0.14 (pb2)  -52.33±0.14 (pb3) |
| PPE:ShPI-1/K13L | -59.12±0.66 | -12.31±0.01 | -6.38±0.59 (2)  -3.08±0.58 (5)  -3.81±0.62 (8)  -4.78±0.57 (pb1)  -3.19±0.58 (pb2)  -2.61±0.60 (pb3) | -18.70±0.59 (2)  -15.39±0.58 (5)  -16.13±0.62 (8)  -17.09±0.56 (pb1)  -15.5±0.58 (pb2)  -14.92±0.60 (pb3) | -77.82±0.12 (2)  -74.51±0.14 (5)  -75.25±0.11 (8)  -76.21±0.19 (pb1)  -74.62±0.14 (pb2)  -74.04±0.13 (pb3) |
| HNE:ShPI-1 | 229.08±0.80 | -11.55±0.01 | -291.19±0.73 (2)  -287.27±0.73 (5)  -291.80±0.73 (8)  -307.78±0.74 (pb1)  -290.03±0.72 (pb2)  -285.06±0.74 (pb3) | -302.75±0.79 (2)  -298.82±0.73 (5)  -302.71±0.76 (8)  -319.33±0.74 (pb1)  -301.58±0.72 (pb2)  -296.61±0.74 (pb3) | -73.67±0.13 (2)  -69.74±0.14 (5)  -74.27±0.11 (8)  -90.26±0.20 (pb1)  -72.50±0.17 (pb2)  -67.53±0.15 (pb3) |
| HNE:ShPI-1/K13L | 200.70±0.74 | -10.14±0.01 | -260.43±0.66 (2)  -261.09±0.66 (5)  -258.74±0.70 (8)  -260.29±0.68 (pb1)  -256.71±0.65 (pb2)  -254.60±0.70 (pb3) | -270.57±0.68 (2)  -271.23±0.65 (5)  -268.88±0.68 (8)  -270.43±0.67 (pb1)  -266.85±0.65 (pb2)  -264.74±0.69 (pb3) | -69.87±0.13 (2)  -70.53±0.14 (5)  -68.18±0.11 (8)  -69.73±0.17 (pb1)  -66.15±0.15 (pb2)  -64.04±0.12 (pb3) |

^b^Standard errors (SE) were calculated through the following formula: SE=SD/√N, where SD is the standard deviation and N, the number of statistically independent frames. ^c^The number between parentheses indicates the *igb* value relative to the GB model used for the calculation of Δ*G_GB_* values. The PB model predictions were indicated by pb1, pb2 and pb3, standing for the PB model using Tan and Luo, mbondi2 and mbondi3 atomic radii, respectively. ^d^The following formulas were used to calculate the different energy components: Δ*E_gas_=*Δ*E_el_*+Δ*E_vw_*, Δ*G_solv_*=Δ*G_GB/PB_*+Δ*G_SA_* and Δ*G_eff_=*Δ*E_gas_*+Δ*G_solv_*.
