# Supplementary material for: Polar Desolvation and Position 226 of Pancreatic and Neutrophil Elastases Are Crucial to their Affinity for the Kunitz-Type Inhibitors ShPI-1 and ShPI-1/K13L
Source: PLoS One. 2015 Sep 15;10(9):e0137787. doi: 10.1371/journal.pone.0137787 (PMC4570792; doi:10.1371/journal.pone.0137787)
Supplement: S10 Table — Only those residues for which |ΔG res|≥1.0 kcal/mol and/or |ΔG sc|≥0.5 kcal/mol at least in one of the complexes are shown here. Note that T226 was included to compare its energy contribution wih that of D226 of HNE, even though it does not fulfill the previous conditions. (DOCX) [file pone.0137787.s015.docx]

|  |  | PPE:ShPI-1/K13L | |  | PPE:ShPI-1up | |  |  |  |
| --- | --- | --- | --- | --- | --- | --- | --- | --- | --- |
|  | Residue | **Δ*G_res_* (kcal/mol)** | Δ*G_sc_*  (kcal/mol) |  | **Δ*G_res_* (kcal/mol)** | Δ*G_sc_*  (kcal/mol) |  | **ΔΔ*G_res_*^a^**  **(kcal/mol)** | **ΔΔ*G_sc_*^a^**  **(kcal/mol)** |
| **PPE residues** | V16 | 2.15^d^ | 0.16 |  | 1.27 | 0.10 |  | 0.88 | 0.06 |
|  | Y35 | -0.92 | -0.88 |  | -1.39 | -1.35 |  | 0.47 | 0.47 |
|  | T41 | -4.04 | -2.56 |  | -4.16 | -2.61 |  | 0.12 | 0.05 |
|  | C42 | -0.95 | -0.75 |  | -1.07 | -0.76 |  | 0.12 | 0.01 |
|  | H57 | -6.86 | -6.55 |  | -7.37 | -7.21 |  | 0.51 | 0.66 |
|  | L63 | -0.48 | -0.52 |  | -0.81 | -0.84 |  | 0.33 | 0.32 |
|  | D98 | -0.80 | -0.85 |  | -0.07 | -0.07 |  | -0.73 | -0.78 |
|  | V99 | -1.44 | -1.41 |  | -1.87 | -1.61 |  | 0.43 | 0.20 |
|  | L143 | -1.24 | -1.09 |  | -0.65 | -0.54 |  | -0.59 | -0.55 |
|  | L151 | -1.67 | -1.49 |  | -1.70 | -1.61 |  | 0.03 | 0.12 |
|  | W172 | -1.07 | -1.07 |  | -0.24 | -0.22 |  | -0.83 | -0.85 |
|  | C191 | -0.43 | -0.40 |  | 0.41 | -0.26 |  | -0.84 | -0.14 |
|  | Q192 | -6.61 | -4.10 |  | -4.21 | -3.06 |  | -2.4 | -1.04 |
|  | G193 | -1.94 | 0 |  | -2.05 | 0 |  | 0.11 | 0 |
|  | D194 | -2.84 | -2.18 |  | -1.28 | -1.00 |  | -1.56 | -1.18 |
|  | S195 | -1.05 | 0.42 |  | 0.10 | 0.48 |  | -1.15 | -0.06 |
|  | T213 | 0.25 | 0.11 |  | 0.30 | 0.08 |  | -0.05 | 0.03 |
|  | S214 | -1.60 | -0.05 |  | -0.16 | -0.05 |  | -1.44 | 0 |
|  | F215 | -3.55 | -1.83 |  | -2.91 | -3.49 |  | -0.64 | 1.66 |
|  | V216 | -2.31 | -0.55 |  | -0.94 | -0.08 |  | -1.37 | -0.47 |
|  | R217A | -1.13 | 0.49 |  | 0.29 | 0.88 |  | -1.42 | -0.39 |
|  | T226 | -0.08 | -0.24 |  | 0.14 | 0.01 |  | -0.22 | -0.25 |
| **Inhibitor residues** | K7(P7)^b^ | 1.33 | 1.01 |  | 0.57 | 0.43 |  | 0.76 | 0.58 |
|  | V9(P5) | -1.19 | -0.94 |  | -0.14 | 0.24 |  | -1.05 | -1.18 |
|  | G10(P4) | 1.09 | 0 |  | 0.58 | 0 |  | 0.51 | 0 |
|  | R11(P3) | -4.30 | -1.10 |  | -0.60 | -0.12 |  | -3.7 | -0.98 |
|  | C12(P2) | -5.51 | -2.25 |  | -1.87 | -1.65 |  | -3.64 | -0.60 |
|  | X13(P1)^c^ | -12.37 | -6.79 |  | -1.82 | 2.12 |  | -10.55 | -8.91 |
|  | G14(P1’) | -4.57 | 0 |  | -5.31 | 0 |  | 0.74 | 0 |
|  | Y15(P2’) | -7.15 | -4.24 |  | -6.87 | -4.69 |  | -0.28 | 0.45 |
|  | F16(P3’) | -5.67 | -4.76 |  | -5.89 | -4.80 |  | 0.22 | 0.04 |
|  | P17(P4’) | -0.84 | -0.63 |  | -0.79 | -0.65 |  | -0.05 | 0.02 |
|  | R18(P5’) | 1.65 | 1.69 |  | 1.32 | 1.34 |  | 0.33 | 0.35 |
|  | I32(SL) | -1.44 | -1.70 |  | -0.64 | -0.89 |  | -0.8 | -0.81 |
|  | Y33(SL) | -0.40 | -1.09 |  | -0.50 | -0.83 |  | 0.1 | -0.26 |
|  | G35(SL) | -1.77 | 0 |  | -0.06 | 0 |  | -1.71 | 0 |
|  | C36(SL) | -3.07 | -2.21 |  | -3.07 | -1.97 |  | 0 | -0.24 |
|  | E44 | 1.18 | 1.09 |  | 1.39 | 1.26 |  | -0.21 | -0.17 |

^a^ΔΔ*G_res(sc)_=*Δ*G_res(sc)_*(PPE:ShPI-1/K13L)-Δ*G_res(sc)_*(PPE:ShPI-1up).

^b^The sites corresponding to the primary binding loop (Pn-Pn’) and the secondary binding loop (SL) of the inhibitor are detailed between parentheses.

^c^X13 stands for either L13 of K13 depending on the complex.

^d^Standard errors are always less than 5% of the mean energy values (not shown).
