# Supplementary material for: Polar Desolvation and Position 226 of Pancreatic and Neutrophil Elastases Are Crucial to their Affinity for the Kunitz-Type Inhibitors ShPI-1 and ShPI-1/K13L
Source: PLoS One. 2015 Sep 15;10(9):e0137787. doi: 10.1371/journal.pone.0137787 (PMC4570792; doi:10.1371/journal.pone.0137787)
Supplement: S1 Text — (DOCX) [file pone.0137787.s016.docx]

Based on our analysis, the hydrogen bond between V216(**N**) and R11(O) at P3 site of ShPI-1 and ShPI-1/K13L displays a high occupancy value. In fact, this interaction was found in the three S3:P3 interfaces (S3 Table) and it is conserved in other complexes of SPs with canonical inhibitors, thereby suggesting its importance for the binding process. In addition, a hydrogen bond between the carbonyl oxygen (O) of the enzyme’s residue at position 41 (T41 in HNE and F41 in PPE) and the nitrogen (**N**) of Y15 at P2’ site was predicted in all S2’:P2’ interfaces. However, two additional hydrogen bonds comprising these residues (T41 and Y15) were predicted in the PPE:ShPI-1/K13L complex (S3 Table). At the secondary binding loop of both inhibitors, G35 forms a hydrogen bond with either R61 or N61 of PPE and HNE, respectively, although displaying highly-variable occupancies within the four complexes (S3 Table), probably caused by the solvent exposure of the interacting residues.

One of the major differences concerning polar interactions outside the S1:P1 interface involves the guanidino nitrogen atoms (NH1 and NH2) of R11 at the P3 site of ShPI-1/K13L. These atoms form various hydrogen bonds and a salt bridge with the carboxylic oxygen atoms of D98 in the PPE complex (S3 and S4 Table). However, the equivalent residue of HNE (V99) only forms one hydrogen bond with R11(**NH1**) at the P3 site of both ShPI-1 variants (Table 1). Furthermore, the hydrogen bond Q192(**NE2**):C12(O) was predicted in the PPE complex, but the presence of Phe at this position of HNE precludes the formation of a similar interaction in the complexes of the latter enzyme (S3 Table). At the edge of primary binding loop, R18(P5) of both inhibitor variants was predicted to form a hydrogen bond with N61 of HNE, but no equivalent interactions with R61 were found in the PPE complex. Interestingly, based on the occupancy values, N61 preferentially interacts with R18 whereas R61 only forms a hydrogen bond with G35 at secondary binding loop of the inhibitor (S3 Table). The cause of this difference becomes clear after superimposing the representative structures of ShPI-1/K13L in complex with both elastases (S4 Fig.).
