# Supplementary material for: Polar Desolvation and Position 226 of Pancreatic and Neutrophil Elastases Are Crucial to their Affinity for the Kunitz-Type Inhibitors ShPI-1 and ShPI-1/K13L
Source: PLoS One. 2015 Sep 15;10(9):e0137787. doi: 10.1371/journal.pone.0137787 (PMC4570792; doi:10.1371/journal.pone.0137787)
Supplement: S2 Text — (DOCX) [file pone.0137787.s017.docx]

In addition to the S1 subsite, we identified warm/hot-spot residues belonging to S3, S2, S1’, S2’ and S3’ subsites (Fig. 7 and see S2 Table for subsite location). For example, residues F41(S1’/S2’), N61(S2’/S3’), L99B(S3/S2) and I151(S2’) of HNE and Y35(S2’), T41(S1’/S2’), D98(S3), V99(S2/S3), L143(S2’), L151(S2’) and W172(S3) of PPE were predicted as hot-spots at least for the interaction with one inhibitor variant (Fig. 11). Conversely, R217A(S3) has an unfavorable contribution to the binding of PPE to both inhibitor variants (Fig. 11). Finally, R36 of HNE is a hot-spot residue located outside the S3-S3’ subsites (Fig. 11).

Some residues of the primary binding loop other than the P1 site, i.e., R11(P3), Y15(P2’) and F16(P3’), were predicted as hot-spots of both inhibitor variants (Fig. 11). Residues C12(P2) and G14(P1’) also have significant energy contributions according to the *pr*EFED protocol predictions (S8, S9 and S10 Table). To a lesser extent, residues I32(P19’), Y33(P20’), G35(P22’) and C36(P23’) of the secondary binding loop contribute to the interaction with both elastases (Fig. 11 and S8, S9 and S10 Table).
